# Perfluorooctanoic acid (PFOA), perfluorooctane sulfonic acid (PFOS), and perfluorononanoic acid (PFNA) increase triglyceride levels and decrease cholesterogenic gene expression in human HepaRG liver cells

**DOI:** 10.1007/s00204-020-02808-0

**Published:** 2020-06-25

**Authors:** Jochem Louisse, Deborah Rijkers, Geert Stoopen, Aafke Janssen, Martijn Staats, Ron Hoogenboom, Sander Kersten, Ad Peijnenburg

**Affiliations:** 1 Wageningen Food Safety Research (WFSR), Wageningen, The Netherlands; 2 Nutrition, Metabolism and Genomics Group, Division of Human Nutrition and Health, Wageningen University, Wageningen, The Netherlands

**Keywords:** PFASs, HepaRG cells, Transcriptomics, Cholesterol, Triglycerides

## Abstract

Per- and polyfluoroalkyl substances (PFASs) are omnipresent in the environment, food chain, and humans. Epidemiological studies have shown a positive association between serum levels of perfluorooctanoic acid (PFOA) and perfluorooctane sulfonic acid (PFOS), and increased serum cholesterol and, in some cases, also triglyceride levels. However, causality has been questioned, as animal studies, as well as a human trial, showed a decrease in serum cholesterol and no effects or a decrease in plasma triglycerides. To obtain more insight into the effects of PFASs on these processes, the present study investigated the effects of PFOA, PFOS, and perfluorononanoic acid (PFNA) on intracellular triglyceride and cholesterol levels in human HepaRG liver cells. DNA microarray analyses were performed to provide insight into underlying mechanisms. All PFASs induced an increase in cellular triglyceride levels, but had no effect on cholesterol levels. Gene set enrichment analysis (GSEA) of the microarray data indicated that gene sets related to cholesterol biosynthesis were repressed by PFOA, PFOS, and PFNA. Other gene sets commonly affected by all PFAS were related to PERK/ATF4 signaling (induced), tRNA amino-acylation (induced), amino acid transport (induced), and glycolysis/gluconeogenesis (repressed). Moreover, numerous target genes of peroxisome proliferator-activated receptor α (PPARα) were found to be upregulated. Altogether, the present study shows that PFOA, PFOS, and PFNA increase triglyceride levels and inhibit cholesterogenic gene expression in HepaRG cells. In addition, the present study indicates that PFASs induce endoplasmic reticulum stress, which may be an important mechanism underlying some of the toxic effects of these chemicals.

**Electronic supplementary material:**

The online version of this article (10.1007/s00204-020-02808-0) contains supplementary material, which is available to authorized users.

## Introduction

Per- and polyfluoroalkyl substances (PFASs) are man-made chemicals that are extremely persistent and omnipresent in the environment (Wang et al. 2017). PFASs contain at least one fluoro-carbon chain of different lengths with varying chemical groups attached, and have unique chemical and physical characteristics, such as oil and water repellency, high temperature and chemical resistance, and emulsifying/surfactant properties. Because of these favorable properties, they are widely used in various industrial and consumer applications, e.g., firefighting foams, electronics, textiles, food contact materials, and cosmetics. According to OECD (2018), over 4700 PFASs have been identified. The production and use of the best-known and most-studied PFASs, perfluorooctanoic acid (PFOA) and perfluorooctane sulfonate (PFOS), have been restricted globally due to concerns of risks to human health and the environment (ATSDR 2018; EFSA CONTAM Panel 2018). However, even though the use of PFOS and PFOA has been restricted, recent assessments of the European Food Safety Authority (EFSA CONTAM Panel 2018, 2020) state that a considerable proportion of the European population is exposed to higher PFAS levels than the tolerably weekly intake (TWI) values.

EFSA CONTAM Panel (2018) derived TWIs for PFOS and PFOA of, respectively, 13 and 6 ng/kw bw/week, based on the positive association between serum PFAS levels and serum cholesterol. This was observed in numerous epidemiological studies, including those by Steenland et al. (2009), Nelson et al. (2010), and Eriksen et al. (2013), studies that were used to derive BMDL_5_ serum levels of 21–25 ng/mL for PFOS and 9.2–9.4 ng/mL for PFOA. Corresponding daily intakes resulting in these serum levels at adult age were established using PBPK modeling, which were then used to derive the two TWIs. An increase in cholesterol levels was seen as adverse, since it is regarded to be a risk factor for cardiovascular disease. Besides an increase in serum cholesterol, exposure to PFOS and PFOA is also positively associated with an increase in serum triglyceride levels in a number of epidemiological studies (EFSA CONTAM Panel 2018). Interestingly, various rat studies, including those by NTP (2019a, b), have shown that exposure to PFASs decreases rather than increases serum cholesterol and triglyceride levels. A similar observation was made in monkeys treated with PFOS (Seacat et al. 2002). Furthermore, data of a recent study on the kinetics and effects of PFOA in human cancer patients in a phase 1 dose-escalation trial also indicate that PFOA reduces serum cholesterol levels, whereas serum triglyceride levels were unaffected (Convertino et al. 2018). These human data are in line with data from a study using APOE*3-Leiden.CETP mice, a model with a human-like lipoprotein metabolism, in which PFOA decreased plasma triglycerides, total cholesterol, and non-HDL-C, whereas HDL-C was increased (Pouwer et al. 2019). It should be noted that in these studies, the exposure and resulting serum levels are much higher than observed in epidemiological studies. Regarding the contradictory findings on the relation between PFAS exposure and cholesterol levels, Convertino et al. (2018) suggested several confounding factors that may explain the observed associations. After the publication of the EFSA Opinion (EFSA Contam Panel 2018), another potential confounding mechanism related to the enterohepatic cycling of both bile acids and PFASs was brought up (https://www.efsa.europa.eu/sites/default/files/news/efsa-contam-3503.pdf). More studies are needed to investigate the underlying mechanisms to support a causal relationship, as also recommended by EFSA in its recent risk assessment (EFSA CONTAM Panel 2020). It is noteworthy that in this new risk assessment, a group TWI of 8 ng/kg bw per week for the sum of four PFASs (PFOA, PFNA, PFHxS, and PFOS) was proposed, based on the effects on the immune system that were also associated with rather low serum levels of PFASs.

In light of the uncertainties around the relation between PFAS exposure and cholesterol and triglyceride levels, a better understanding of how PFASs may interfere with cholesterol and triglyceride metabolism is thus required. Inasmuch as the liver plays an important role in the regulation of cholesterol and triglyceride levels, mechanistic in vitro studies with human liver cells are important to gain insight into the effects of PFASs on these processes. In animals, PFASs have been shown to cause hypertrophy and hyperplasia of the liver, but also steatosis and possibly cholestasis (EFSA CONTAM Panel 2018, 2020). To obtain more insight into the effects of PFASs on the human liver, the effects of PFOS and PFOA and the related PFAS perfluorononanoic acid (PFNA) in human HepaRG liver cells were determined in the present study. Effects on PFAS-induced changes in cellular cholesterol and triglyceride levels were assessed, followed by transcriptomics analysis to gain insight into possible underlying mechanisms.

## Materials and methods

### Chemicals

**Fig. 1:**
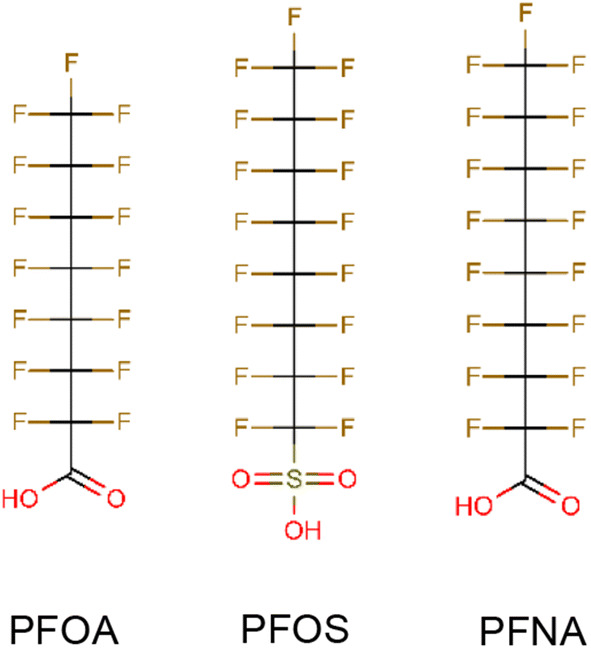
Chemical structures of the PFASs tested in the present study

PFOA (purity 99%) and PFNA (purity 99%) were purchased from Sigma-Aldrich (Zwijndrecht, The Netherlands) and PFOS (purity 100%) was obtained from Synquest laboratories (Alachua FL). All stock solutions (dilution series) of the compounds were prepared in 100% dimethyl sulfoxide (DMSO HybriMax, Sigma-Aldrich). Chemical structures of the three PFASs are shown in Fig. 1.

### HepaRG cell culture

The human hepatic cell line HepaRG was obtained from Biopredic International (Rennes, France) and cultured in growth medium consisting of William’s Medium E+GlutaMAX™ (ThemoFisher Scientific, Landsmeer, The Netherlands) supplemented with 10% Good Forte filtrated bovine serum (FBS; PAN™ Biotech, Aidenbach, Germany), 1% PS (100 U/mL penicillin, 100 µg/mL streptomycin; Capricorn Scientific, Ebsdorfergrund, Germany), 50 µM hydrocortisone hemisuccinate (sodium salt) (Sigma-Aldrich), and 5 µg/mL human insulin (PAN™ Biotech). Seeding, trypsinization [using 0.05% Trypsin–EDTA (ThermoFisher Scientific)], and maintenance of the cells was performed according to the HepaRG instruction manual from Biopredic International. For cell viability studies, HepaRG cells were seeded in black-coated 96-well plates (Greiner Bio-One, Frickenhausen, Germany; 9000 cells per well in 100 µL). For gene expression studies and analysis of triglyceride and cholesterol levels, HepaRG cells were seeded in 24-well plates (Corning, Corning, NY; 55,000 cells per well in 500 µL). After 2 weeks on growth medium, cells were cultured for two days in growth medium supplemented with 0.85% DMSO to induce differentiation. Subsequently, cells were cultured for 12 days in growth medium supplemented with 1.7% DMSO (differentiation medium) for final differentiation. At this stage, cells were ready to be used for toxicity studies. Cells that were not immediately used were kept on differentiation medium for a maximum of 3 additional weeks. Cell cultures were maintained in an incubator (humidified atmosphere with 5% CO_2_ at 37 °C) and the medium was refreshed every 2–3 days during culturing. Prior to toxicity studies, differentiated HepaRG cells were incubated for 24 h in assay medium (growth medium-containing 2% FBS) supplemented with 0.5% DMSO.

### Cell exposure

Test chemicals were diluted from 200-fold concentrated stock solutions in the assay medium, providing a final DMSO concentration of 0.5%. In each experiment, a solvent control (0.5% DMSO) was included. PFASs were tested in concentrations up to 400 µM. Different exposure durations and concentrations were used. After exposure, the effects of the PFASs on cell viability, triglyceride, and cholesterol levels, and gene expression were assessed.

### Cell viability studies

The effect of the chemicals on cell viability was in the first instance determined on PFAS-exposed HepaRG cells cultured in 96-well plates, using the WST-1 assay. This assay determines the conversion of the tetrazolium salt WST-1 (4-[3-(4-iodophenyl)-2-(4-nitrophenyl)-2*H*-5-tetrazolio]-1,3-benzene disulfonate) to formazan by metabolically active cells. After exposure for 6, 24, or 72 h, the medium was removed and the cells were washed with Dulbecco’s Phosphate-Buffered Saline (DPBS; ThermoFisher Scientific). Next, WST-1 solution (Sigma-Aldrich) was added to the cell culture medium (1:10 dilution) and 100 µL was added to each well. After 1 h incubation in an incubator (humidified atmosphere with 5% CO_2_ at 37 °C), the plate was shaken at 1000 rpm for 1 min, and absorbance at 450 nm was measured (background absorbance at 630 nm was subtracted) using a microplate reader (Synergy™ HT BioTek, Winooski, VT, USA). Further experiments (cholesterol and triglyceride analyses and gene expression analyses) were performed in 24-well plates. Therefore, possible effects on cell viability were also examined in HepaRG cells in 24-well plates, by determining the total DNA quantity in each well. To that end, cells were lysed with RLT-lysis buffer (Qiagen, Venlo, The Netherlands) and the Quant-iT™ PicoGreen^®^ dsDNA Assay Kit (Life Technologies) was used for DNA quantification. The samples were diluted two times in DPBS. Samples were also diluted another two times (in 1:1 RLT:DPBS). TE reagent was prepared according to the manufacturer’s protocol. A standard curve of dsDNA in TE reagent was prepared; a range of 1.25–0.002 µg/mL was used for quantification. In a black 96-well plate, 5 µL sample and 195 µL TE reagent were added, protected from light. DPBS:RLT (1:1) was taken along as a blanc. The plate was mixed in the dark for 4 min at 1100 rpm, and afterwards incubated for 5 min at room temperature. Fluorescence was measured using a microplate reader (Synergy™ HT BioTek) at excitation 485/20 nm, emission 528/20 nm, and a sensitivity of 40.

### Triglyceride and cholesterol analysis

To determine the effect of PFAS exposure on triglyceride and cholesterol levels, a method based on gas chromatography with flame-ionization detection (GC-FID) was applied. To this end, differentiated HepaRG cells in 24-well culture plates were exposed for 24 h to PFOS, PFOA, or PFNA at concentrations ranging from 25 to 200 µM. After exposure, cells were washed three times with 0.5 mL DPBS/well. Cells were harvested in 150 µL RLT-lysis buffer and lysates of two wells were pooled in 1.5 mL Eppendorf Protein LoBind microcentrifuge tubes (Fisher Scientific, Hampton, USA). The samples were stored at − 80 °C until extraction. The extraction of triglycerides was performed under an N_2_ atmosphere according to a method described by Hutchins et al. (2008) using isooctane:ethyl acetate (75:25). After thawing, the samples were transferred to 10 mL glass tubes. A mixture of isooctane and ethyl acetate (75:25, 5 mL) with 10 µL 0.5 mg/mL tritridecanoin (Nu-Chek Prep Inc., Elysian, USA) was added to the samples, followed by vortex-mixing (30 s), sonication (60 s), vortex-mixing (30 s), and incubation head over head (15 min). NaCl (1 mL, 0.9% w/v) was added and the samples were vortex-mixed for 60 s and centrifuged for 10 min at 1000×*g* to separate the two phases. A total of 4.2 mL of the organic (upper) phase were collected with a 1-mL glass Hamilton syringe. The polar phase was extracted again by adding 2 mL isooctane:ethyl acetate (75:25) followed by vortex-mixing, centrifugation, and collection of the organic phase. The two organic phases were combined, dried under N_2_ gas at 30–37 ℃, redissolved in 100 µL isooctane, and transferred to a GC vial. The samples were analyzed on a Trace GC Ultra GC-FID system (ThermoFisher Scientific, Waltham, USA). A sample volume of 2 µL was injected on a 5 m × 0.53 mm, 0.17 µm Sim Dist Ulti Metal column (CP7532, Agilent Technologies, Santa Clara, USA) and eluted at a N_2_ gas flow of 45.5 mL/min. The temperature program was as follows: 0.5 min 80 °C, 50 °C/min ramp to 190 °C, 6 °C/min ramp to 350 °C, and 5 min hold at 350 °C. The detector temperature was 370 °C. A triglyceride standard mixture (palm kernel triglycerides, BCR 632 A, Sigma-Aldrich, St. Louis, USA) was included in each sequence to calibrate the retention times of the triglycerides. Pure cholesterol (Sigma-Aldrich) was used to calibrate the retention time of cholesterol. Triglycerides (C44, C46, C48, C50, C52, and C54) and cholesterol were quantified by determining the AUC. The relative triglyceride and cholesterol levels were calculated by dividing the AUCs of triglycerides and cholesterol obtained upon treatment with the test compounds by the AUCs of triglycerides and cholesterol obtained with the solvent control sample. Statistical differences were assessed by performing a one-way ANOVA followed by Dunnett's test on the normalized data (fold-change compared to the solvent control) using Graphpad Prism 5, considering a *p* value < 0.05 as being statistically significant. The concentration–response data were also used for BMD analysis as described below.

### Whole-genome gene expression analysis: microarray hybridisations and analysis

To obtain insight into the molecular and cellular effects of PFASs in the liver, differentiated HepaRG cells were exposed for 6, 24, or 72 h to 100 µM PFOA and for 24 h to 100 µM PFOS or 100 µM PFNA. After exposure, total RNA was isolated and purified using the RNeasy Minikit (Qiagen). RNA quality and integrity were assessed using the RNA 6000 Nano chips on the Agilent 2100 Bioanalyzer (Agilent Technologies, Amsterdam, The Netherlands). Purified RNA (100 ng) was labeled with the Ambion WT expression kit (Invitrogen) and hybridized to Affymetrix Human Gene 2.1 ST arrays (Affymetrix, Santa Clara, CA). Hybridization, washing, and scanning were carried out on an Affymetrix GeneTitan platform according to the instruction by the manufacturer. Quality control analysis, array normalization, and statistical analyses were carried out using MADMAX (Lin et al. 2011). For array normalization, the Robust Multiarray Average method (Bolstad et al. 2003; Irizarry et al. 2003) was applied. Probe sets were defined according to Dai et al. (2005). In this method, probes are assigned to Entrez IDs as a unique gene identifier. Subsequently, data were filtered with IQR 0.25 and three arrays with signal > 20. *p* values for the effect of the PFAS treatments were calculated using an Intensity-Based Moderated T-statistic (IBMT) (Sartor et al. 2006). Significantly differentially expressed genes (DEGs) were selected using a *p* value of < 0.001 and fold-change of > 1.5 (treatment versus DMSO control) as cut-offs. Hierarchical clustering of DEGs was performed using the coolmap function (R/coolmap.R) in limma. The log-expression values were prepared by subtracting from each value the mean log2(FC) of the DMSO controls. Clustering of these control-corrected log2(FC) expression values was performed using complete linkage and Euclidean distance. Pathway analysis on DEGs was performed using ConsensusPathDB (CPDB). CPDB analysis was applied using the webtool https://cpdb.molgen.mpg.de, which combines and compares the results of multiple pathway databases (Kamburov et al. 2011; Herwig et al. 2016). For the analysis in the present study, four databases were selected, i.e., Reactome, KEGG, Wikipathways, and Biocarta. Pathways with a *p* value < 10^−4^ were considered to be affected significantly.

In a next step, MADMAX was used to perform a gene set enrichment analysis (GSEA) of the normalized and filtered data to identify enriched gene sets (Subramanian et al. 2005). Using this approach, genes are ranked based on the paired IBMT statistics and subsequently analyzed for over- or underrepresentation in predefined gene sets derived from Gene Ontology, KEGG, National Cancer Institute, PFAM, Biocarta, Reactome, and WikiPathways pathway databases. Only gene sets consisting of more than 15 and fewer than 500 genes were taken into account. Statistical significance of GSEA results was determined using 1000 permutations.

### Real-time qPCR

For selected genes, concentration-dependent expression levels were determined in HepaRG cells. To that end, HepaRG cells were exposed to increasing concentrations of PFOA, PFOS, or PFNA for 24 h and total RNA was extracted from the HepaRG cells using the RNeasy Mini Kit (Qiagen, Venlo, The Netherlands). Subsequently, 500 ng RNA was used to synthesize cDNA using the iScript cDNA synthesis kit (Bio-Rad Laboratories, Veenendaal, The Netherlands). Changes in gene expression were determined by real-time PCR on a CFX384 real-time PCR detection system (Bio-Rad Laboratories) using SensiMix (Bioline; GC Biotech, Alphen aan den Rijn, The Netherlands). The PCR conditions consisted of an initial denaturation of 95 °C for 10 min, followed by 40 cycles of denaturation at 95 °C for 10 s and annealing extension at 60 °C for 15 s. The housekeeping gene *RPL27* was used for normalization. *RPL27* was chosen, since this has been reported to be one of the most stable genes based on a meta-analysis of 13,629 human gene array samples to identify the most stable expressed genes (de Jonge et al. 2007). The more commonly used beta-actin and/or GAPDH vary considerably under different experimental conditions (de Jonge et al. 2007). Primer sequences were taken from the Harvard PrimerBank and ordered from Eurogentec (Liège, Belgium). Sequences of the used primers are listed in Table 1. Statistical differences were assessed by performing a one-way ANOVA followed by Dunnett's test on the normalized data (fold-change compared to the solvent control) using Graphpad Prism 5, considering a *p* value < 0.05 as being statistically significant. The concentration–response data were also used for benchmark dose (BMD) analysis as described below.

**Table 1:** Primer sequences used for qPCR

Gene name	Primer sequence	
	Forward	Reverse
*RPL27*	ATCGCCAAGAGATCAAAGATAA	TCTGAAGACATCCTTATTGACG
*ANGPTL4*	CACAGCCTGCAGACACAACTC	GGAGGCCAAACTGGCTTTGC
*PDK4*	TGGAGCATTTCTCGCGCTAC	ACAGGCAATTCTTGTCGCAAA
*PLIN2*	ATGGCATCCGTTGCAGTTGAT	GATGGTCTTCACACCGTTCTC
*PLIN4*	GGCACCAAGAACACTGTCTG	TCGTACCCATGACCATAGACTT
*CPT1A*	TCCAGTTGGCTTATCGTGGTG	CTAACGAGGGGTCGATCTTGG
*ADH4*	AGTTCGCATTCAGATCATTGCT	CTGGCCCAATACTTTCCACAA
*LSS*	GCACTGGACGGGTGATTATGG	TCTCTTCTCTGTATCCGGCTG
*FDPS*	CTCCTCCCTCAGAATGAACG	CACCCTAACGATCTGGGAGA
*HMGCR*	TGATTGACCTTTCCAGAGCAAG	CTAAAATTGCCATTCCACGAGC
*EBP*	CTCAGCACCTAAGACTGGACA	ACGACTAAGACCCCTGTGACA
*IDI1*	TCCATTAAGCAATCCAGCCGA	CCCAGATACCATCAGACTGAGC
*ACAT2*	CCCAGCCAATGCTTCAGGAAT	AAGCCCACGTTTATCAGCTTC

### BMD analysis of qPCR and cellular triglyceride data

To obtain more insight into possible differences in potencies of the three PFASs regarding their effects on triglyceride and cholesterol levels and on their effects on the expression of selected genes, concentration–response modeling and benchmark concentration analysis were performed using the PROAST webtool (PROASTweb version 65.2, RIVM, Bilthoven, The Netherlands, https://proastweb.rivm.nl), essentially as recommended by EFSA (EFSA Scientific Committee 2017). PROAST is particularly applied for modeling of in vivo (dose–response) data, providing information on the benchmark dose (BMD). We used the PROAST software for the analysis of in vitro (concentration–response) data, thereby providing information on the benchmark concentration (BMC). Tab-delimited text files containing data on concentration, mean effect (normalized effect to the solvent control), standard deviation, and sample size (number of biological replicates) were uploaded to the PROAST webtool and analyzed as continuous (summary) data. In the tool, data are fitted to a number of mathematical models. The models showing the best (goodness of) fit, i.e., having the lowest Akaike Information Criterion (AIC) value, were used for calculation of the BMC and the corresponding two-sided 90% BMC confidence interval given by the BMCL (lower bound of the BMC confidence interval) and the BMCU (upper bound of the BMC confidence interval). The BMC, BMCL, and BMCU were determined for a benchmark response of 50% (BMR_50_) which corresponds to a 50% increase over the background response, resulting in a BMC_50_, BMCL_50_, and BMCU_50_. In PROAST, the used definitions are CES (critical effect size), CED (critical effect dose), CEDL (lower bound of the CED), CEDU (upper bound of the CED), which are identical to BMR, BMC, BMCL, and BMCU, respectively.

## Results

### Cell viability studies

**Fig. 2:**
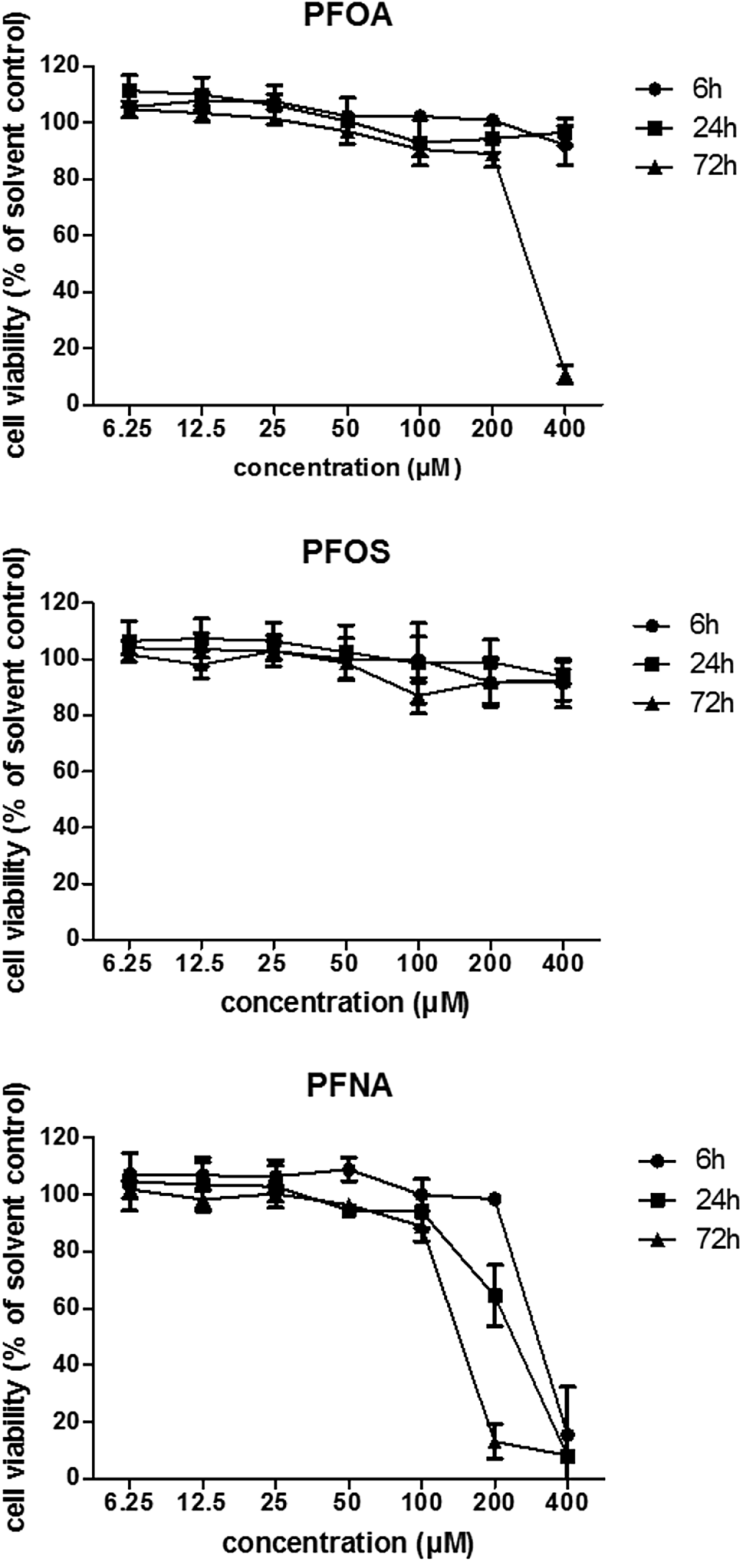
Effects of 6, 24, and 72 h exposure to PFOA, PFOS, or PFNA on viability of HepaRG cells as determined with the WST-1 assay and expressed as % of the solvent control (0.5% DMSO). Data presented as mean ± SD of three independent experiments (using per independent experiment the mean of three technical replicates)

In the first instance, the WST-1 assay was performed to get an impression on the PFAS concentrations that affect the viability of HepaRG cells. To that end, HepaRG cells in 96-well plates were exposed for 6, 24, and 72 h to increasing concentrations (up to 400 µM) of PFOA, PFOS, and PFNA. PFOA did not decrease cell viability upon 6 or 24 h exposure at any of the concentrations tested, but showed a clear drop in cell viability upon 72 h exposure at 400 µM (Fig. 2). PFOS did not show a decrease in cell viability (not larger than 20%) in any of the treatments (6, 24, or 72 h; Fig. 2). PFNA was the most cytotoxic PFAS, causing a more than 20% decrease in cell viability upon exposure for 6 h (400 µM), 24 h (200 and 400 µM) and 72 h (200 and 400 µM) (Fig. 2). Cell viability upon a 24-h exposure was also determined in 24-well plates by quantifying the amount of DNA per well. These studies indicated that DNA quantity was unaffected up to 200 µM PFOA, 100 µM PFOA or 100 µM PFNA (data not shown). Therefore, these concentrations were applied as maximum concentrations in the further studies.

### Triglyceride and cholesterol levels in cells

**Fig. 3:**
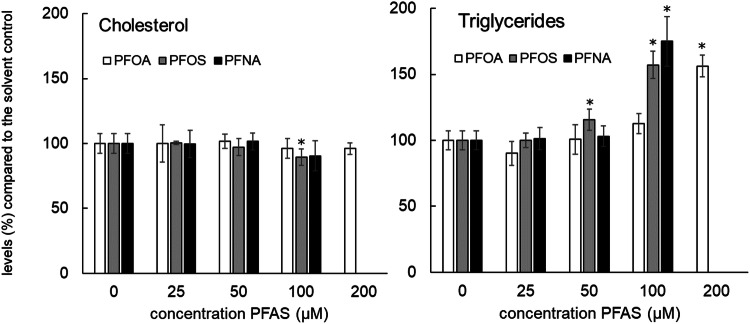
Cholesterol and triglyceride levels in HepaRG cells exposed for 24 h to increasing concentrations of PFOA, PFOS, and PFNA. Cholesterol and triglyceride levels were normalized to the levels as measured in the solvent control, which was set at 100%. Treatments with a statistically significant difference (*p* < 0.05) in triglyceride or cholesterol levels compared to the solvent control are indicated with *. Data presented as mean ± SD of 4 (treatments) to 12 (controls) replicates obtained in two independent experiments

Concentration-dependent effects of PFASs on triglyceride and cholesterol levels were determined by performing an extraction of HepaRG cells that were exposed for 24 h to increasing (non-cytotoxic) PFAS concentrations, followed by detection using GC-FID. The outcome of these analyses is presented in Fig. 3. All PFASs caused a concentration-dependent increase in cellular triglyceride levels. Regarding the effects of the PFASs on cholesterol levels, limited effects were noticed. A slight decrease was observed at 100 µM PFOS and PFNA, but these effects were only statistically significant for PFOS. Dose–response modeling on the triglyceride data was performed using BMD analysis to obtain more insight into potency differences (Supplementary Fig. 1). BMC_50_ values (Table 2) indicate that PFOS and PFNA are more potent than PFOA.

**Table 2:** BMC_50_ values of PFAS-induced increase in triglycerides (sum of C44, C46, C48, C50, C52, and C54)

	BMC_50_ (µM)	
	Expon model	Hill model
PFOA	184 (167–201)	184 (167–201)
PFOS	93 (86–101)	93 (86–101)
PFNA	93 (86–96)	92 (86–96)

BMC_50_ values are given in µM and BMCL–BMCU range is presented in brackets. Data are presented for the Exponential (Expon) model and the Hill model. Related concentration–response curves are presented in Supplementary Fig. 1

### Microarray analysis: effects of 24 h exposure to 100 µM PFASs

**Fig. 4:**
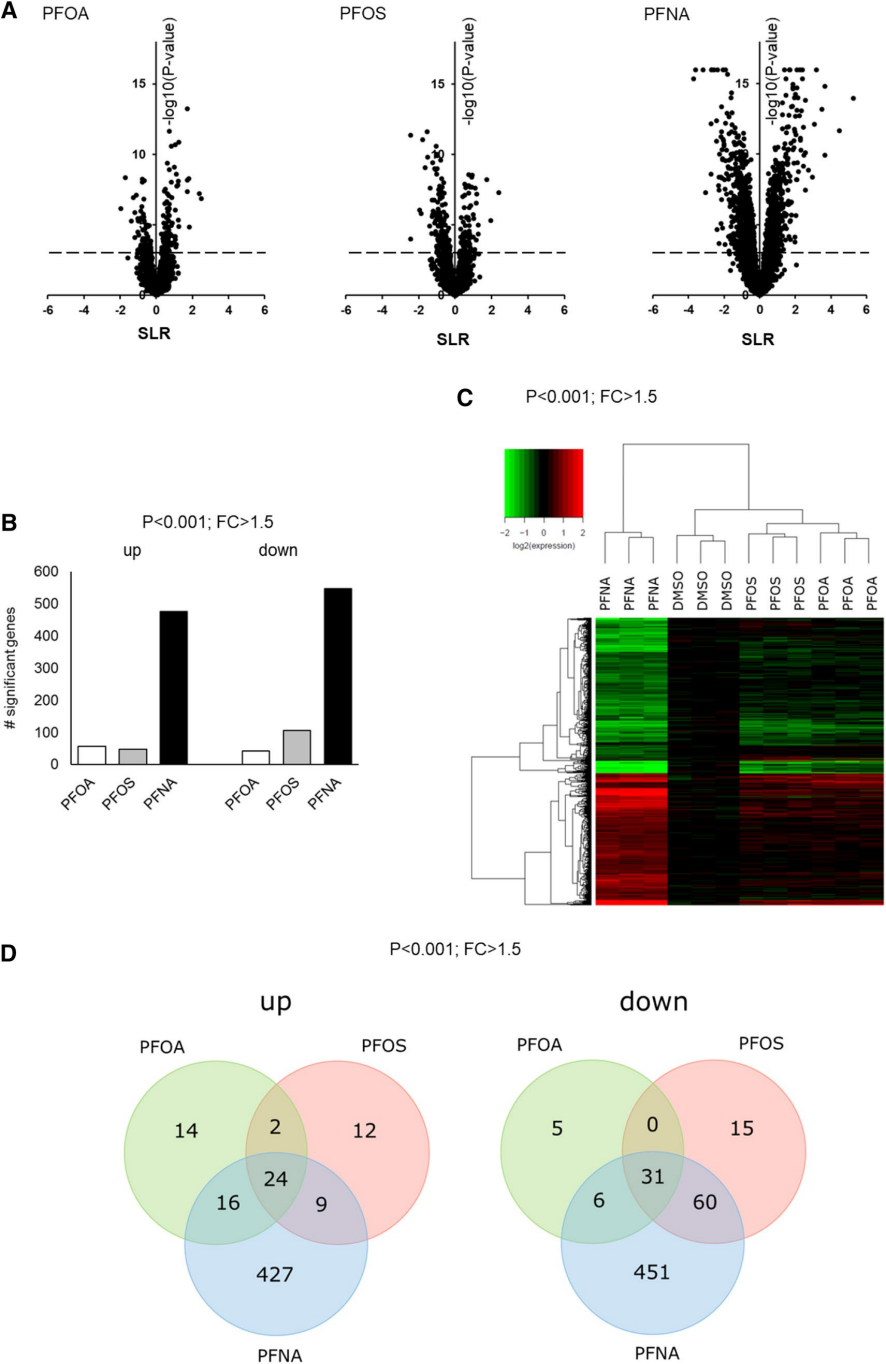
Effects of PFOA, PFOS, and PFNA on whole-genome gene expression in HepaRG cells, exposed for 24 h to 100 µM PFOA, PFOS or PFNA. **a** Volcano plots showing relative changes in gene expression (expressed as signal log(2) ratio (SLR), *x*-axis) plotted against statistical significance (expressed as − log10 *p* value of IBMT regularized paired *t* test, *y* axis). Dotted line represents cut-off of *p* < 0.001. **b** The number of up- or downregulated genes based on a statistical significance cut-off of *p* < 0.001 (IBMT regularized paired *t* test) and a fold-change (FC) > 1.5. **c** Heatmap obtained upon a hierarchical clustering of DEGs. **d** Venn diagrams showing the number of genes up- and downregulated by the three PFASs. Material was obtained from three independent experiments

Since none of the PFASs were cytotoxic at 100 µM, this concentration was used to study the effect of PFASs on whole-genome gene expression. HepaRG cells were exposed for 24 h to 100 µM PFOA, PFOS, or PFNA, and subjected to DNA microarray analysis. For PFOA, also a time-course experiment was executed in which HepaRG cells were treated with 100 µM PFOA for 6, 24, or 72 h followed by gene expression profiling. Figure 4 shows an overview of the results of the microarray data of HepaRG cells exposed for 24 h to 100 µM PFOA, PFOS, or PFNA. Upon normalization and filtering of the array data, Volcano plots were generated, which show that PFNA had the largest effect on gene expression in HepaRG cells (Fig. 4a). Subsequently, differentially expressed genes (DEGs) were selected using a *p* value of < 0.001 (IBMT regularized paired *t* test) and fold-change of > 1.5 (treatment versus DMSO control) as cut-offs (for DEGs and corresponding *p*- and fold-change values, see Supplementary Table 1). Applying these cut-offs, PFOA significantly modulated the expression of 98 genes of which 56 genes were upregulated and 42 genes were downregulated. The expression of 153 genes was found to be significantly altered by PFOS treatment (47 genes upregulated and 106 genes downregulated). The most pronounced effect was observed for the PFNA treatment (1024 DEGs), resulting in 476 upregulated and 548 downregulated genes (Fig. 4b). Upon hierarchical clustering of the in total 1069 DEGs, PFNA treatment is clearly separated from the other treatments as indicated in the heatmap, as shown in Fig. 4c. Venn diagrams of the upregulated (left) and downregulated (right) genes (Fig. 4d) show that 24 genes are commonly upregulated by PFOA, PFOS, and PFNA, and that 31 genes are commonly downregulated by these three PFASs. The expression of these genes is presented in Supplementary Fig. 2.

A concise pathway analysis was performed on these common DEGs using ConsensusPathDB (CPDB). For the 24 upregulated genes, the top 3 enriched pathway-based sets were “Amino acid synthesis and interconversion (transamination)” (Reactome), “Serine biosynthesis” (Reactome), and “PPAR signaling pathway (human)” (KEGG). For the downregulated genes, the three most significantly affected pathways were “Glycolysis/Gluconeogenesis (human)” (KEGG), “Urea cycle” (Reactome), and “Ethanol oxidation” (Reactome). More detailed results on the CPDB analysis of commonly modulated genes, as well as genes specifically regulated by one or two of the PFASs, are presented in Supplementary Table 2. Overall, the largest effects on gene expression changes were observed for PFNA, followed by PFOS and PFOA.

**Fig. 5:**
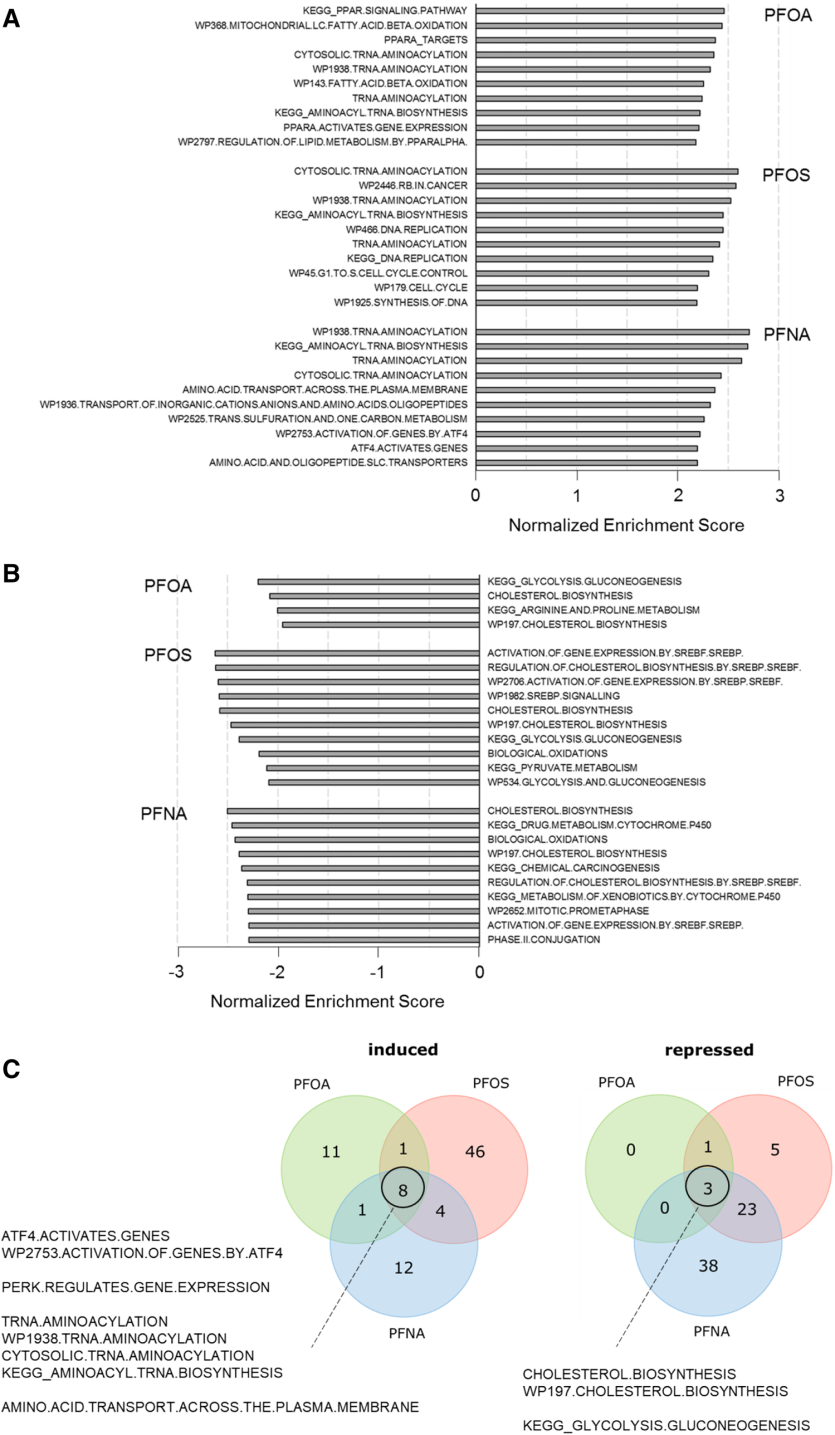
The top ten most strongly induced (**a**) or repressed (**b**) gene sets in HepaRG cells in response to a 24-h exposure to PFOA, PFOS or PFNA, as determined on the basis of the normalized enrichment score (NES) and statistical significance (FDR *q* value < 0.05) obtained with the gene set enrichment analysis (GSEA). For PFOA, only four gene sets were found to be repressed. **c** Venn diagram showing the number of gene sets induced (positive NER) and repressed (negative NER) by PFOA, PFOS, and PFNA, describing gene sets commonly affected by the three PFASs. Only gene sets were included with an FDR *q* value < 0.05

To obtain a better and more detailed insight into the biological pathways regulated by PFOA, PFOS, and PFNA in HepaRG cells, gene set enrichment analysis (GSEA) was performed. Figure 5 presents the top ten gene sets from these analyses for each PFAS, based on the obtained normalized enrichment scores (NERs) of significantly regulated gene sets (FDR *q* value < 0.05). Gene sets related to “PPAR signaling”, “lipid metabolism”, “fatty acid beta oxidation”, and “tRNA amino-acylation” featured prominently among the gene sets induced by PFOA (Fig. 5a). Gene sets induced by PFOS were related to “tRNA amino-acylation”, as well as to “RB (retinoblastoma protein) pathway in cancer”, and “cell cycle” (Fig. 5a). For PFNA, induction of genes related to “tRNA amino-acylation”, and “amino acid/oligopeptide transport” was observed (Fig. 5a). Only four gene sets were significantly repressed by PFOA, which are related to “cholesterol biosynthesis”, “arginine and proline metabolism”, and “glycolysis/gluconeogenesis” (Fig. 5b). “Cholesterol biosynthesis” and “glycolysis/gluconeogenesis” were also among the gene sets most prominently repressed by PFOS (Fig. 5b). PFNA repressed gene sets mainly related to “cholesterol biosynthesis” and “xenobiotics metabolism” (Fig. 5b).

The number of significantly affected gene sets by the three PFASs and the overlapping gene sets are visualized in the Venn diagram presented in Fig. 5c. When comparing all significantly regulated (*q* < 0.05) gene sets, it was found that PFOA, PFOS, and PFNA affect a number of common gene sets, including gene sets related to cholesterol biosynthesis (repressed), glycolysis/gluconeogenesis (repressed), tRNA amino-acylation (induced), amino acid transport across the cell membrane (induced), gene expression regulation by ATF4 (induced), and gene regulation by PERK (induced; large overlap with gene sets related to gene expression regulation by ATF4).

**Fig. 6:**
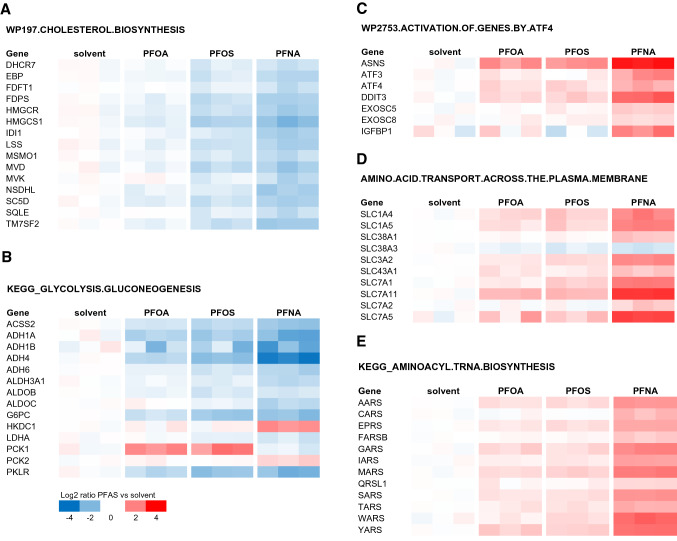
PFAS-induced changes in expression of genes related to cholesterol biosynthesis (from Reactome; **a**), glycolysis/gluconeogenesis (from Kegg; **b**), PERK/ATF signaling (from Wikipathways; **c**), amino acid transport across the cell membrane (from Reactome; **d**), and aminoacyl-tRNA biosynthesis (from Kegg; **e**). Only genes are shown for which at least one of the PFASs induced a significant change in gene expression with at least a 1.5-fold change in expression. Genes were grouped according to the overlapping gene sets identified in the GSEA (Fig. 5c)

Figure 6 shows the expression of genes related to the gene sets that were commonly repressed (Fig. 6a, b) or induced (Fig. 6c–e) by the three PFASs. As shown in Fig. 6a, the majority of the genes belonging to the gene set cholesterol biosynthesis were downregulated, showing the largest effects for PFNA, followed by PFOS, and suggesting the lowest potency for PFOA. The GSEA also indicated that the gene set related to glycolysis/gluconeogenesis was repressed (Fig. 6b). Several genes involved in glycolysis, gluconeogenesis, or both, were downregulated, whereas *PCK1* was upregulated by PFOA and PFOS, and *PCK2* and *HKDC1* by PFNA. This gene set (KEGG_GLYCOLYSIS.GLUCONEOGENESIS) also contains several downregulated genes involved in ethanol metabolism, one of the commonly affected pathways identified with the CPDB analysis. As shown in Fig. 6c–e, the expression of genes related to ATF4-related gene expression, amino acid transport across the cell membrane, and aminoacyl-tRNA biosynthesis was upregulated, showing the largest effects for PFNA, while similar effects were observed for PFOS and PFOA.

### Comparison of PFAS-induced gene expression with gene expression data of a typical PPARα-agonist (GW7647), LXR-agonist (GW3965), and FXR-agonist (CDCA)

**Fig. 7:**
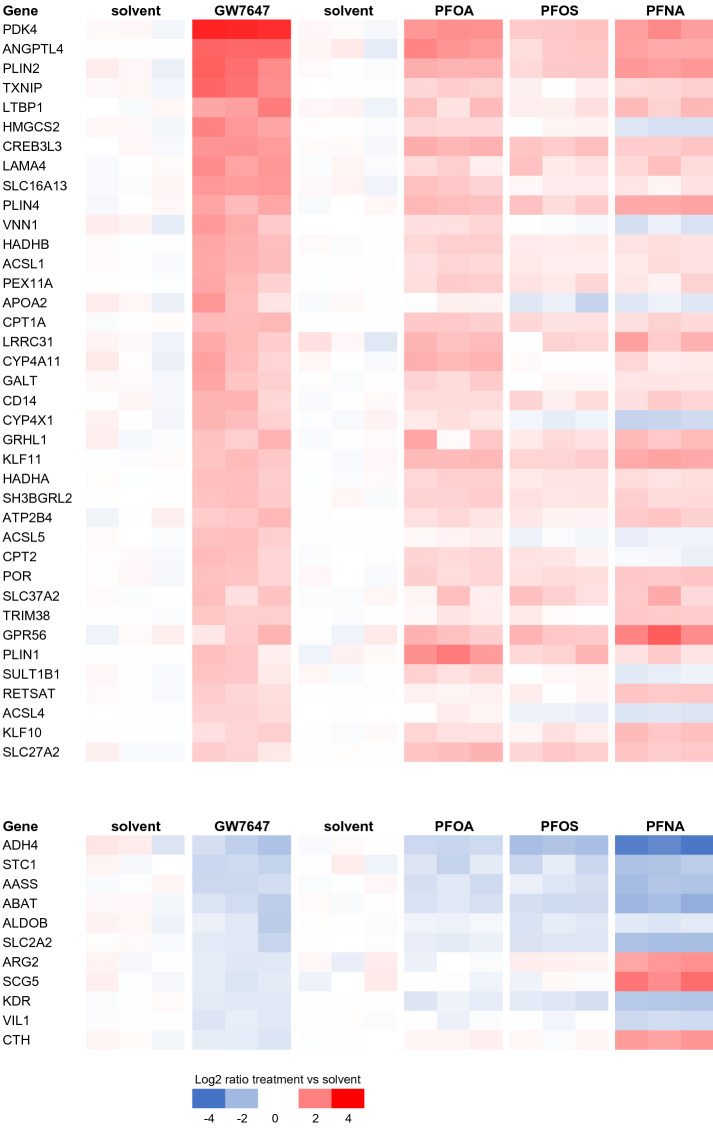
Gene expression changes induced by PPARα-agonist GW7647 (data from Wigger et al. 2019) compared with gene expression changes induced by PFASs (data from the present study) in HepaRG cells. GW7647-related DEGs for which at least one of the PFASs has a DEG are shown. Expressions of the solvent controls from the respective studies are shown left of the treatment data

Peroxisome proliferator-activated receptor α (PPARα) is considered to be a relevant target of PFASs and was, indeed, one of the pathways identified upon CPDB analysis and for PFOA by the GSEA (see above). To assess whether the PFAS-induced expression profiles resemble the expression profile induced by a typical PPARα-agonist, we used the microarray data from the study of Wigger et al. (2019), who exposed HepaRG cells for 4 and 24 h to a series of nuclear receptor (NR) agonists [available on GEO (accession number: GSE124053)]. The data were analyzed using MADMAX as described above for the PFAS microarray data, to obtain lists of DEGs related to a 24-h exposure to GW7647 (PPARα-agonist), but also GW3965 [liver X receptor (LXR)-agonist] and the bile acid CDCA [farnesoid X receptor (FXR)-agonist]. Comparing these DEGs with the DEGs related to PFAS exposure indicates that the PFASs, indeed, show a gene expression profile similar to the gene expression profile induced by the PPARα-agonist GW7647. Typical genes include *PDK4*, *ANGPTL4*, *CPT1A,* and *CYP4A11*. This applies in particular to PFOA and PFNA, but less for PFOS (Fig. 7).

Gene expression profiles induced by the LXR-agonist GW3965 and FXR-agonist CDCA show a few similarities with the gene expression profiles induced by the PFASs (Supplementary Figs. 3, 4). Salient is that the gene most prominently downregulated by CDCA, *CYP7A1*, is also downregulated by all three PFASs, particularly by PFNA. This gene is known to be involved in the conversion of cholesterol to bile acids. *CYP3A4* was upregulated by both the LXR-agonist GW3965 and FXR-agonist CDCA, as well as by the PFASs, and is involved in bile acid metabolism and xenobiotic metabolism.

We also assessed the effects of the three model NR agonists on the expression of genes within the gene sets commonly affected by the PFASs (Supplementary Fig. 5). These analyses indicate that the effects of model NR agonists on these genes are in general different from the effects of the PFASs.

### Microarray analysis: effects of 6, 24, or 72 h exposure to PFOA

In addition to the analysis of a 24-h exposure to PFOA, PFOS, or PFNA, a time-course experiment was conducted in which HepaRG cells were exposed for 6, 24, or 72 h to 100 µM PFOA, followed by DNA microarray analysis. After 6 h exposure, the number of significantly regulated genes was relatively low compared to 24 h, especially for the downregulated genes (Supplementary Fig. 6). After 72 h of exposure, the number of significantly regulated genes was slightly higher than after 24 h, albeit differences were small (Supplementary Fig. 6). GSEA revealed that gene sets related to “PPARα signaling”, “regulation of lipid metabolism by PPARα”, “(mitochondrial) fatty acid beta oxidation”, and “fatty acid degradation” were significantly induced at all time points (Supplementary Fig. 7). Only one and four gene sets were significantly repressed upon 6 and 24 h exposure, respectively, and no gene set was repressed upon a 72 h exposure (Supplementary Fig. 7).

### Gene expression analysis of selected genes related to PPAR signaling and cholesterol biosynthesis

**Fig. 8:**
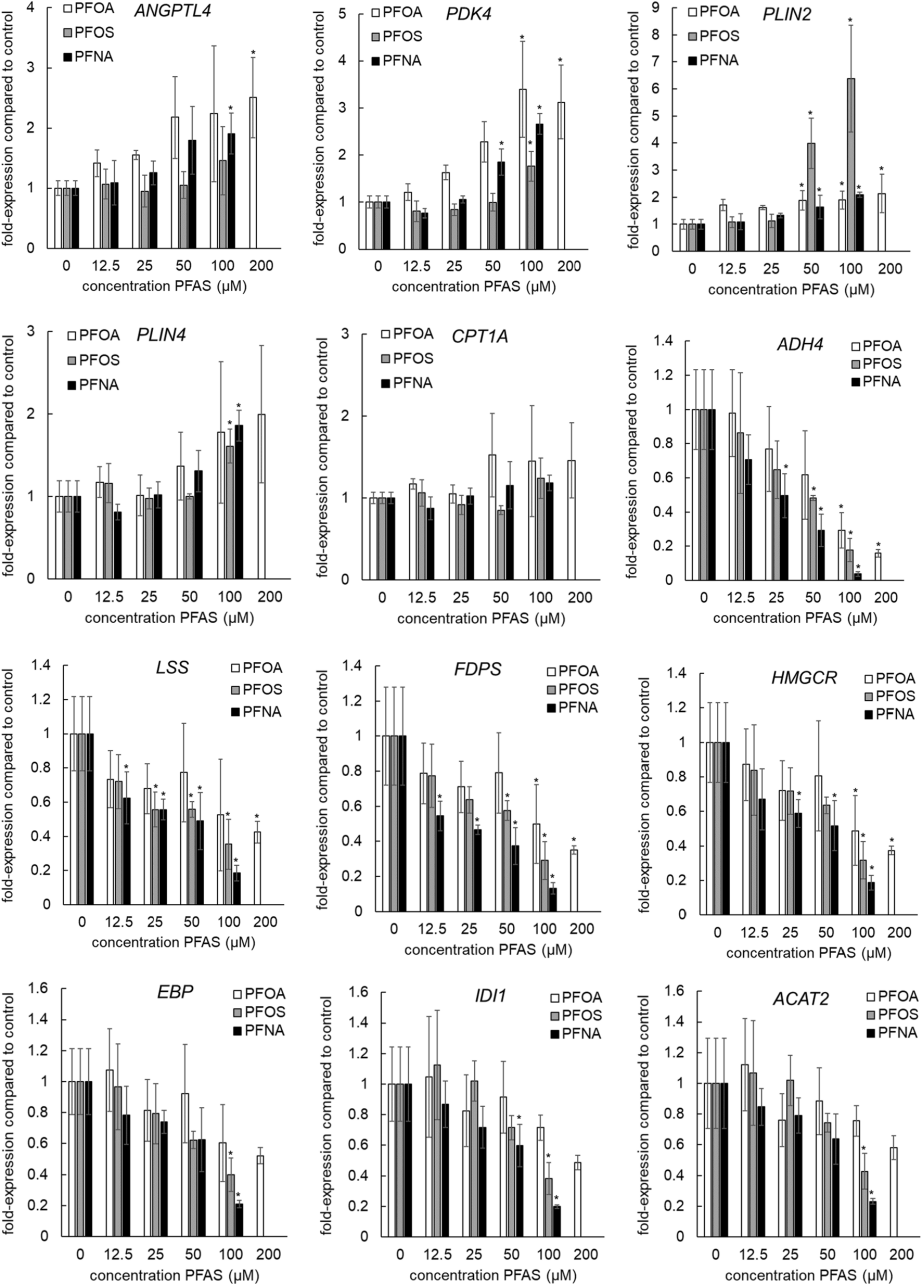
Concentration-dependent effects on gene expression of the PPAR-responsive genes (*ANGPTL4*, *PDK4*, *PLIN2*, *PLIN4*, and *CPT1A*), *ADH4*, and genes related to cholesterol biosynthesis (*LSS*, *FDPS*, *HMGCR*, *EBP*, *IDI1*, and *ACAT2*) by PFOS, PFOA, and PFNA. Gene expression was normalized to the housekeeping gene *RPL27* and the gene expression of the solvent control was set at 1. Treatments with statistically significant difference (*p* < 0.05) in gene expression compared to the solvent control are indicated with * (one-way ANOVA followed by Dunnett’s multiple comparison test (Graphpad Prism 5). Data presented as mean ± SD of three independent experiments

To gain some insight into potency differences between PFOA, PFOS, and PFNA in the PFAS-induced PPAR activation and inhibition of cholesterol biosynthesis, concentration-dependent gene expression of five selected PPARα target genes (*ANGPTL4*, *PDK4, PLIN2*, *PLIN4,* and *CPT1A*), *ADH4* [the gene most downregulated by the PPARα agonist GW7647, and also strongly downregulated by the PFASs (Fig. 7)] and six selected genes related to cholesterol biosynthesis (*FDPS*, *LSS*, *HMGCR*, *EBP*, *IDI1,* and *ACAT2*) was determined using qPCR analysis. Concentration-dependent increases in the expression of *PLIN2*, *PLIN4,* and *PDK4* were observed for the three tested PFASs, whereas *ANGPTL4* expression only significantly increased upon PFOA and PFNA exposure. Effects on *CPT1A* were limited: only a small increase was noticed upon PFOA exposure. *ADH4* was downregulated by the three PFASs, already at relatively low concentrations, showing the highest potency for PFNA and a similar potency for PFOA and PFOS (Fig. 8). Concentration-dependent decreases in the expression of *FDPS, LSS*, *HMGCR*, *EBP*, *IDI1,* and *ACAT2* were observed for all three PFASs, although not statistically significant for *IDI1* and *ACAT2* upon PFOA exposure (Fig. 8). Concentration–response modeling using BMD analysis was performed on the normalized expression data to calculate BMC_50_ values (Supplementary Fig. 8). Obtained BMC_50_ values (Table 3) suggest that PFOA is the most potent in activating the PPARα response genes followed by PFNA, although potency differences between the PFASs appeared to be gene-specific. Interestingly, *PLIN2* expression was most induced by PFOS, although the BMC50 concentration was lower for PFOA. Regarding effects on the expression of cholesterogenic genes, PFNA and PFOS showed to be more potent inhibitors than PFOA (Table 3).

**Table 3:** BMC_50_ values of PFAS-induced changes in gene expression of PPAR-responsive genes (*ANGPTL4*, *PDK4*, *PLIN2*, *PLIN4*, and *CPT1A*), *ADH4*, and genes related to cholesterol biosynthesis (*LSS*, *FDPS*, *HMGCR*, *EBP*, *IDI1*, and *ACAT2*)

	BMC50 (µM)											
	*ANGPTL4*	*PDK4*	*PLIN2*	*PLIN4*	*CPT1A*	*ADH4*	*LSS*	*FDPS*	*HMGCR*	*EBP*	*IDI1*	*ACAT2*
PFOA												
Expon model	19 (6.7–43)	20 (13–29)	11 (7.1–14)	77 (19–173)	174 (28–5190)	56 (38–74)	130 (30–621)	131 (54–215)	118 (53–198)	181 (96–357)	198 (135–306)	> 200
Hill model	18 (7.3–41)	19 (13–89)	11 (7.0–18)	77 (19–173)	173 (28–5100)	50 (30–75)	131 (29–626)	131 (54–215)	118 (53–199)	181 (97–355)	198 (135–307)	> 200
PFOS												
Expon model	NA	72 (51–91)	32 (24–35)	92 (67–108)	> 100	58 (33–78)	40 (8.9–310)	79 (33–99)	85 (55–101)	89 (54–122)	81 (65–94)	88 (71–103)
Hill model	NA	72 (51–91)	28 (24–32)	92 (67–108)	> 100	58 (33–78)	40 (8.7–315)	79 (33–99)	85 (55–101)	89 (54–123)	81 (65–94)	88 (71–103)
PFNA												
Expon model	44 (14–89)	34 (28–41)	42 (21–67)	55 (37–74)	> 100	33 (24–42)	43 (21–69)	28 (14–49)	52 (29–74)	68 (52–81)	63 (49–75)	71 (57–84)
Hill model	44 (14–89)	33 (27–39)	42 (21–67)	47 (33–65)	> 100	33 (24–42)	43 (211–69)	28 (14–49)	53 (29–74)	68 (52–81)	63 (49–75)	71 (57–84)

BMC_50_ values are given in µM and BMCL–BMCU range is presented in brackets. Data are presented for the Exponential (Expon) model and the Hill model. Related concentration–response curves are presented in Supplementary Fig. 8

*NA* not applicable: no dose–response and thus BMC_50_ obtained

## Discussion

The present study assessed the effects of PFOA, PFOS, and PFNA on human HepaRG liver cells, with a particular interest in the effects of these compounds on cholesterol and triglyceride metabolism. All three PFASs downregulated cholesterogenic genes, with PFNA being the most potent and PFOA the least potent. Despite these changes, none of the PFASs significantly changed cellular cholesterol levels. All three PFASs caused an increase in cellular triglyceride levels, with PFOS and PFNA having similar potency and PFOA having a somewhat lower potency. Other cellular processes affected by the PFASs, as indicated by the gene expression analyses, point to effects on PERK/ATF4 signaling, tRNA amino-acylation, amino acid transport, and glycolysis/gluconeogenesis, and support the activation of PPARα.

The expression of cholesterogenic genes decreased upon treatment with all three PFASs. At 100 µM, the strongest effects were observed for PFNA and PFOS (Fig. 6). The relatively low potency of PFOA was confirmed by concentration–response analysis of qPCR data of six selected genes of the cholesterol biosynthesis pathway, i.e., *LSS*, *FDPS*,* HMGCR*, *EBP*, *IDI1,* and *ACAT2*. This qPCR analysis also suggested PFNA to be slightly more potent than PFOS in inhibiting cholesterogenic gene expression. A decrease in expression of genes related to cholesterol synthesis was also shown by Behr et al. (2020b) for PFOA and PFOS in HepaRG cells. Cellular cholesterol biosynthesis is regulated by intracellular cholesterol levels, which is mediated by sterol regulatory element-binding proteins (SREBPs) 1 and 2 (Shimano 2001; Horton et al. 2003; Adams et al. 2004; DeBose-Boyd and Ye 2018). SREBPs are synthesized as inactive precursors and held in a tripartite complex with SREBP cleavage-activating protein (SCAP) and INSIG-1 in the endoplasmic reticulum (ER) membrane. In response to low cholesterol levels, INSIG-1 dissociates and the SCAP–SREBP complex is translocated to the Golgi apparatus for proteolytic activation. The resulting active N‐terminal SREBP enters the nucleus, where it binds to sterol regulatory elements in the promoter regions of target genes, including genes involved in cholesterol synthesis. In the presence of sufficient cholesterol, cholesterol accumulates in the ER membrane, causing a conformational change in SCAP resulting in binding of SCAP to INSIG-1, release of COPII, and trapping of SCAP in the ER membranes. As a result, SREBP is not transported to the Golgi and will not be activated. Besides cholesterol, unsaturated fatty acids have been reported to prevent SREBP transport from the ER to the Golgi, thereby inhibiting SREBP signaling, which may result in a decrease in cholesterol synthesis. Unsaturated fatty acids may stabilize INSIG-1 in the ER membrane, causing SCAP (and SREBP) to remain in the ER (DeBose-Boyd and Ye 2018). Interestingly, gene sets related to the regulation of cholesterol biosynthesis by SREBP (e.g., Wikipathway 1982) were downregulated, especially by PFNA and PFOS (Fig. 5b; Supplementary Fig. 9), suggesting that the PFASs inhibit SREBP-mediated gene expression in HepaRG cells. Accordingly, it can be hypothesized that PFASs may mimic the suppressive effect of unsaturated fatty acids on SREBP signaling.

Various NRs have been reported to play a role in the control of cholesterol homeostasis, such as PPARs, LXRs, and FXRs (Ory 2004; Li and Glass 2004; Li and Chiang 2009). Several studies have assessed whether PFASs bind and activate (or inhibit) gene expression regulated by different NRs (from different species), e.g., using reporter gene systems. From these studies, it is evident that various PFASs induce PPARα-mediated gene expression, and that activation of other NRs, including the ERα, ERβ, AR, CAR, FXR, LXRα, LXRβ, PPARβ, PPARγ, RARα, and RXRα, seems to be limited (Vanden Heuvel et al. 2006; Wolf et al. 2014; Behr et al. 2018, 2020a). Comparison of the gene expression data obtained upon HepaRG exposure to PFASs from the present study with those obtained after HepaRG exposure to a PPARα-agonist (GW7647), an LXR-agonist (GW3965) and an FXR-agonist (CDCA) reported by Wigger et al. (2019), indicate that the PFASs act as PPARα-agonists, but not as FXR nor as LXR agonists, corroborating the data obtained in the reporter gene systems. It can, therefore, be concluded that the tested PFASs in our study, indeed, activate PPARα-mediated gene expression, especially PFOA and PFNA, and, to a lesser extent, PFOS (Fig. 7). The relatively low activity of PFOS compared to PFOA and PFNA has also been shown by Wolf et al. (2014) using a reporter gene assay with human PPARα.

Various endogenous ligands have been reported for PPARα, including fatty acids, various eicosanoids, oxidized phospholipids, and oleoylethanolamide (Grygiel-Gorniak 2014; Schupp and Lazar 2010). Activation of PPARα is well known to promote fatty acid oxidation (Dreyer et al. 1993), which is expected to lead to a reduction in intracellular triglyceride levels. Consistent with this notion, PPARα deficiency in mice leads to a fatty liver (Costet et al. 1998; Kersten et al. 1999). By contrast, in the present study, all PFASs induced a concentration-dependent increase in triglyceride levels, suggesting that the triglyceride-raising effect is independent of PPARα. Increased liver triglyceride levels have also been observed in laboratory animals treated with PFASs, including PPARα knockout mice (Das et al. 2017). However, rather than only promoting fatty acid catabolism, PPARα also induces the expression of numerous genes involved in triglyceride synthesis and storage, including several lipid droplet-associated proteins of the PLIN family. Indeed, PFASs induced the expression of the PPARα target genes PLIN2 and PLIN4, with strong induction by all three PFASs (Fig. 8, Table 3). Accordingly, it is conceivable that the induction of PLIN2 and/or PLIN4 mediates the observed increase in cellular triglycerides by PFASs. The notion that different PPARα agonists may have different potencies towards different target genes is called the SPPARM (selective PPAR modulator) concept (Fruchart and Santos 2019) and may explain why certain PPARα target genes are most strongly induced by one PFAS, whereas others are most strongly induced by another.

Treatment with PPARα agonists is known to lower plasma triglyceride levels. Because of this property, a number of PPARα agonists are in clinical use for the treatment of hypertriglyceridemia and atherogenic dyslipidemia. Given that PFASs and in particular PFOA and PFNA are potent PPARα activators, this group of compounds may also impact on plasma triglyceride levels. Indeed, PFASs were previously shown to markedly reduce plasma triglycerides in mice by stimulating lipoprotein lipase-mediated triglyceride clearance and by inhibiting the secretion of triglycerides and ApoB by the liver (Bijland et al. 2011). Intriguingly, some epidemiological studies suggest that exposure to PFASs is associated with increased rather than decreased plasma triglyceride levels (e.g., Steenland et al. 2009; Zeng et al. 2015). Whether these data reflect a causal relationship between PFASs and triglycerides remains unclear.

Epidemiological data have also revealed a positive association between plasma levels of PFASs and serum cholesterol levels (Steenland et al. 2009; Nelson et al. 2010; Eriksen et al. 2013). Currently, it is unclear whether this association reflects a causal effect of PFASs on plasma cholesterol or whether it is due to confounding. The downregulation of genes involved in cholesterol biosynthesis, also observed by Behr et al. (2020b), seems to contradict the positive association between serum cholesterol and PFASs. Nevertheless, it is interesting to speculate on potential causal mechanisms. Intracellular cholesterol levels are tightly regulated (Luo et al. 2020). Therefore, if cholesterol levels in hepatocytes would increase, these are expected to be excreted into bile (via ABCG5/8) and/or into the circulation (via VLDL particles). Hypothetically, this may result in increased serum cholesterol levels. We did not measure cholesterol levels in the medium of the cell cultures, so we cannot exclude such an effect. Behr et al. (2020b) recently measured cholesterol levels in PFOS- or PFOA-exposed HepaRG cells and the cell culture medium, using the fluorescence-based AmplexRed Cholesterol Assay. They did not find changes in cellular cholesterol levels, but reported a small increase of cholesterol in the medium upon a 24-h exposure to 100 µM PFOS, but not at lower concentrations, nor by PFOA. Another hypothesis on a possible mechanism underlying increased serum cholesterol levels can be based on the data presented here, in which we find a marked repression of the SREBP pathway by PFOS and PFNA (Supplementary Fig. 9), including downregulation of the LDL receptor gene (*LDLR*). It can be hypothesized that PFASs may be able to raise plasma cholesterol by suppressing SREBP-dependent transcription of *LDLR*, thus decreasing the hepatocellular uptake of cholesterol-rich LDL, which may result in an increase in serum cholesterol levels. Future studies should be directed towards further investigating the effect of PFASs on LDL receptor protein levels in human hepatocytes.

Although the three PFASs showed, in general, a different gene expression profile than the bile acid CDCA, similarities were also observed (Supplementary Fig. 4). *CYP7A1*, which codes for the key regulatory enzyme in the synthesis of bile acids from cholesterol, was found to be the gene most downregulated by CDCA in HepaRG cells (Wigger et al. 2019). *CYP7A1* was also downregulated by the three PFASs in our study as well as by PFOA and PFOS in the HepaRG study of Behr et al. (2020b). It is of interest to note that Behr et al. (2020b) also observed changes in cellular and extracellular bile acid composition, and that, dependent on the concentration and the chemical (PFOA or PFOS), cholic acid (CA) levels increased (GCA and TCA), whereas chenodeoxycholic acid (CDCA) levels decreased (GCDCA and TCDCA). At the gene expression level, our study shows a decrease in the expression of *CYP8B1* and *CYP27A1* upon PFNA treatment (Supplementary Table 1), playing a role in CA and CDCA formation, respectively. Bile acids can regulate their synthesis via a feedback mechanism by activating FXR, which results in increased expression of the gene *NR0B2* encoding small heterodimer partner (SHP), leading to a decrease in *CYP7A1* expression (Chiang 2009). However, in our study, *NR0B2* expression was downregulated by PFOS and PFNA (Supplementary Table 1), as also reported by Behr et al. (2020b) for PFOA and PFOS and the *CYP7A1* downregulation is, therefore, not expected to be mediated via FXR/SHP. Behr et al. (2020b) suggested *CYP7A1* downregulation to be mediated via downregulation of *HNF4A* (based on Abrahamsson et al. (2005)), but *HNF4A* was only significantly downregulated in our study by PFNA, not fully supporting this mechanism. In agreement with the data of Behr et al. (2020b), we found, besides the downregulation of *CYP7A1*, also an upregulation of *CYP3A4*, which both have been described as key events in the AOP for cholestasis, and, thus, may contribute to the development of cholestasis (Vinken et al. 2013).

Gene sets related to glycolysis/gluconeogenesis were downregulated by all three PFASs, as indicated by the GSEA (Fig. 6b). However, *PCK1*, which codes for a key enzyme in gluconeogenesis (catalyzing the formation of P-enolpyruvate from oxaloacetate), was upregulated by PFOA and PFOS. PFNA induced an upregulation of *PCK2*, which is a mitochondrial variant of *PCK1*. *PKLR*, involved in the glycolysis (conversion of P-enolpyruvate to pyruvate), and *G6PC*, covering the last step in gluconeogenesis (conversion of glucose-6P to glucose), were downregulated by all 3 PFASs. In addition, the expression of *PDK4* was strongly upregulated, which may result in decreased activity of pyruvate dehydrogenase linking glycolysis with the citric acid cycle. When considering the changes in expression of these genes, the resulting cellular effects may point to the process of glyceroneogenesis (Hanson and Reshef 2003; Reshef et al. 2003; Nye et al. 2008). The upregulation of *PCK1* (and possibly also *PCK2*) and downregulation of *PKLR* may result in increased P-enolpyruvate levels, suggesting the process to be directed to gluconeogenesis. However, given the downregulation of the aldolases (*ALDOB* and *ALDOC*), glyceraldehyde 3-P conceivably is not directed towards fructose-1,6-diphosphate but towards dihydroxyacetone-P, which can be used for triglyceride synthesis. Glyceroneogenesis was first described in white adipose tissue, but has also been reported to take place in the liver (Reshef et al. 2003) and is supported by the increased levels of cellular triglyceride levels in the present study.

The gene expression data from our study also point to disturbance of the endoplasmic reticulum (ER) membrane by the three PFASs, reflected by the activation of PERK/ATF4 signaling (Fig. 6c) (Liu et al. 2015; Rozpedek et al. 2016). ER stress induces Unfolded Protein Response (UPR) pathways via activation of protein kinase RNA-like endoplasmic reticulum kinase (PERK) and subsequent phosphorylation of Eukaryotic Initiation Factor 2 alpha (eIF2α). This activation of eIF2α results in attenuation of global protein translation and triggers preferential translation of selected genes such as Activating Transcription Factor 4 (ATF4). ATF4 upregulates genes that play a role in cell recovery, adaptation to stress conditions, and restoration of cell homeostasis (Rozpedek et al. 2016), including genes that are involved in cell metabolism and nutrient transport. Interestingly, it has been demonstrated that during ER stress, e.g., caused by amino acid starvation, ATF4 induced the expression of aminoacyl-tRNA synthetase (ARS) genes and genes coding for amino acid transporters (Adams 2007; Shan et al. 2016; Krokowski et al. 2013; Han et al. 2013). These genes were found to be upregulated by the PFASs in the present study (Fig. 6d, e). Upon continued stress conditions, PERK/ATF4 may also activate downstream *CHOP* (= *DDIT3*; Fig. 6c), which promotes apoptosis (Rozpedek et al. 2016). *CHOP*/*DDIT3* expression was highly induced by PFNA (Fig. 6c). Activation of these pathways has also been reported to be induced by saturated fatty acids in human liver cells, possibly playing a role in the induction of non-alcoholic fatty liver disease (NAFLD) (Cao et al. 2012). Previously, it has been demonstrated that PFOA induces ER stress and UPR in HepG2 liver cells (Yan et al. 2015) and ER-stress-related autophagy in A549 lung cells (Xin et al. 2018).

It is of interest to note that the concentrations applied in the present study (µM range) are relatively high compared to the serum concentrations that have been reported to relate to an increase in serum cholesterol in the epidemiological studies (nM range), especially considering differences in the free fraction of the PFASs in vitro (relatively high free fraction) versus in vivo (relatively low free fraction). Therefore, although the present study provides insight into mechanisms underlying PFAS toxicity, the effects as determined in the present study do not necessarily take place in humans at relevant exposure levels. Therefore, it would be of interest to test in the future the effects upon chronic exposure to PFASs at lower concentrations, as this may better resemble relevant human exposure. Interestingly, reported internal concentrations of PFOA in human cancer patients in a phase 1 dose-escalation trial were in the µM to mM range (Convertino et al. 2018), being in the range of concentrations tested in the present study. The reported decrease in serum cholesterol levels in these patients with increasing PFOA concentrations (Convertino et al. 2018), may relate to the decrease in cholesterogenic gene expression as identified in the present study.

 In conclusion, the results of the present study point to an increase in cellular triglycerides upon PFAS exposure in HepaRG cells and a decrease in cholesterogenic gene expression. The latter is unlikely to be the indirect consequence of increased cholesterol levels, since our GC-FID analyses showed no effects of PFASs on cellular cholesterol levels. Rather, the effect may be due to PFAS-induced inhibition of SREBP signaling, as has been reported before for unsaturated fatty acids (Cao et al. 2012). Furthermore, although PFOA, PFNA, and, to a lesser extent, PFOS activated PPARα signaling, an increase rather than a decrease in cellular triglyceride levels was observed in PFAS-exposed HepaRG cells. The mechanisms underlying this PFAS-induced increase in triglyceride levels remain to be unravelled, but may be related to induction of specific lipid droplet-associated proteins or to a possible induction of glyceroneogenesis. Interestingly, the PFASs also induced expression of genes related to ER disturbance, activating PERK-eIF2α-ATF4 signaling, which may explain the upregulation of aminoacyl-tRNA biosynthesis and amino acid transporters. Altogether, the present study provides more insight into the molecular effects of PFASs on triglyceride levels and cholesterogenic gene expression in human liver cells, but can neither confirm nor contradict a possible causality between PFAS exposure and increased serum cholesterol as identified in human epidemiological studies.

## Electronic supplementary material

Below is the link to the electronic supplementary material.

Supplementary file1 (DOCX 1803 kb)

Supplementary file2 (XLSX 83 kb)

Supplementary file3 (XLSX 25 kb)
