# Supplementary material for: Perfluorooctanoic acid (PFOA), perfluorooctane sulfonic acid (PFOS), and perfluorononanoic acid (PFNA) increase triglyceride levels and decrease cholesterogenic gene expression in human HepaRG liver cells
Source: Arch Toxicol. 2020 Jun 25;94(9):3137–55. doi: 10.1007/s00204-020-02808-0 (PMC7415755; doi:10.1007/s00204-020-02808-0)
Supplement: Supplementary file 1 — Supplementary file1 (DOCX 1803 kb) [file 204_2020_2808_MOESM1_ESM.docx]

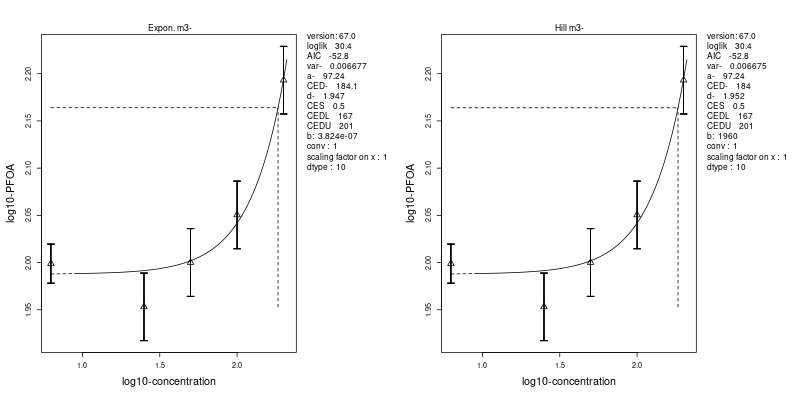

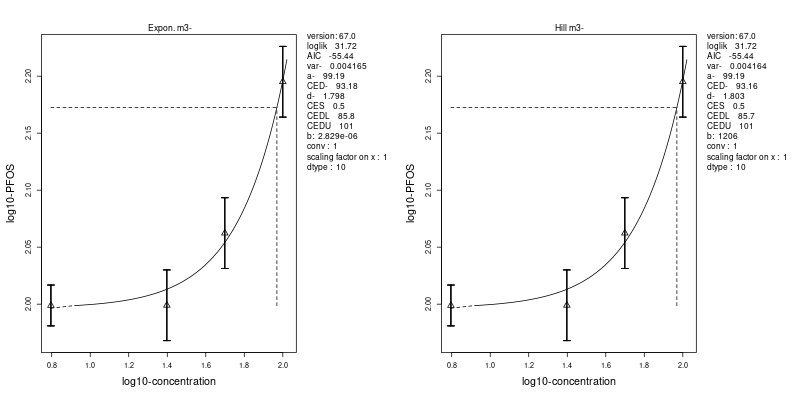

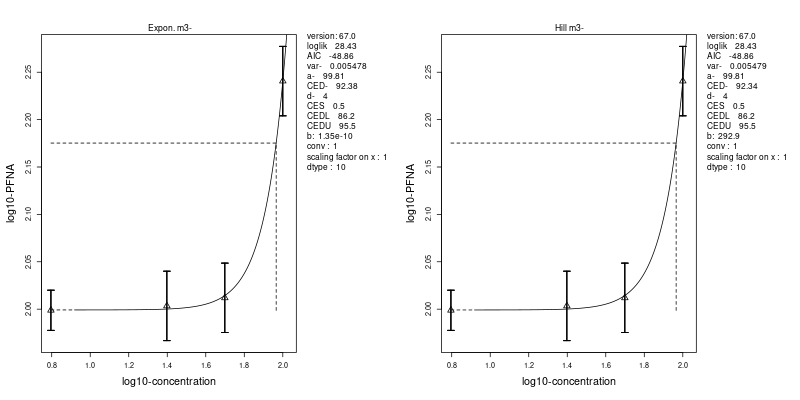


*Supplementary Fig. 1. BMD analysis of changes in cellular triglyceride levels induced by exposure to PFOA (upper), PFOS (middle) and PFNA (lower), using two different curve fits (left: exponential, and right: Hill-function).*

*Supplementary Fig. 2. Common DEGs for PFOA, PFOS and PFNA (24 upregulated and 31 downregulated genes).*

*Supplementary Fig. 3. Gene expression changes induced by LXR-agonist GW3965 (data from Wigger et al. (2019)) compared with gene expression changes induced by PFASs (data from present study) in HepaRG cells. GW3965-related DEGs for which at least one of the PFASs has a DEG are shown. Expression of the solvent controls from the respective studies are shown left of the treatment data.*

*Supplementary Fig. 4. Gene expression changes induced by FXR-agonist CDCA (data from Wigger et al. (2019)) compared with gene expression changes induced by PFASs (data from present study) in HepaRG cells. CDCA-related DEGs for which at least one of the PFASs has a DEG are shown. Expression of the solvent controls from the respective studies are shown left of the treatment data.*

******

******

******

******

******

*Supplementary Fig. 5. GW7647-, GW3965-, CDCA- and PFAS-induced changes in expression of genes related to cholesterol biosynthesis, glycolysis/gluconeogenesis, PERK/ATF signaling, amino acid transport across the cell membrane, and aminoacyl tRNA biosynthesis. GW7647, GW3965 en CDCA data are from Wigger et al. (2019) and PFAS data are from the present study. Only genes are shown for which at least one of the PFASs induced a significant change in gene expression with at least a 1.5-fold change in expression (same genes as presented in Fig. 6). Expression of the solvent controls from the respective studies are shown left of the treatment data.*


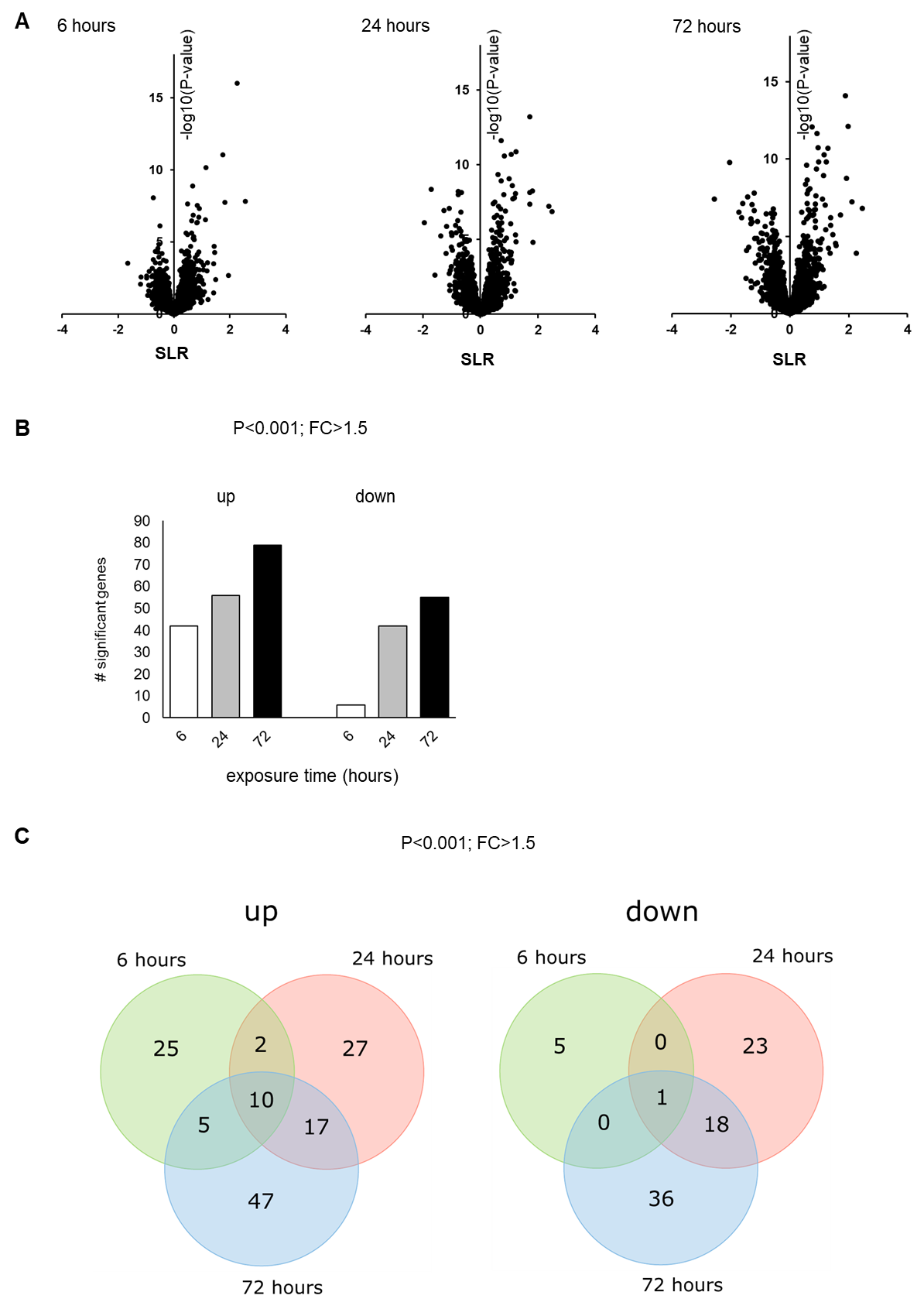


*Supplementary Fig. 6. Analysis of effects of 100 µM PFOA upon 6, 24 or 72 h exposure on whole genome gene expression in HepaRG cells. A) Volcano plots showing relative changes in gene expression in response to a 6-, 24- or 72-h exposure (expressed as signal log(2) ratio (SLR), x-axis) plotted against statistical significance (expressed as –log10 p-value of IBMT regularized paired t-test, y-axis). Dotted line represents cut-off of p<0.001. B) The number of differentially expressed genes (DEGs) in HepaRG cells in response to a 6-, 24- or 72-h exposure based on a statistical significance cut-off of P < 0.001 (IBMT regularized paired t-test) and a fold-change (FC) >1.5. C) Venn diagrams showing the number of up- and downregulated genes at the three time-points.*

*
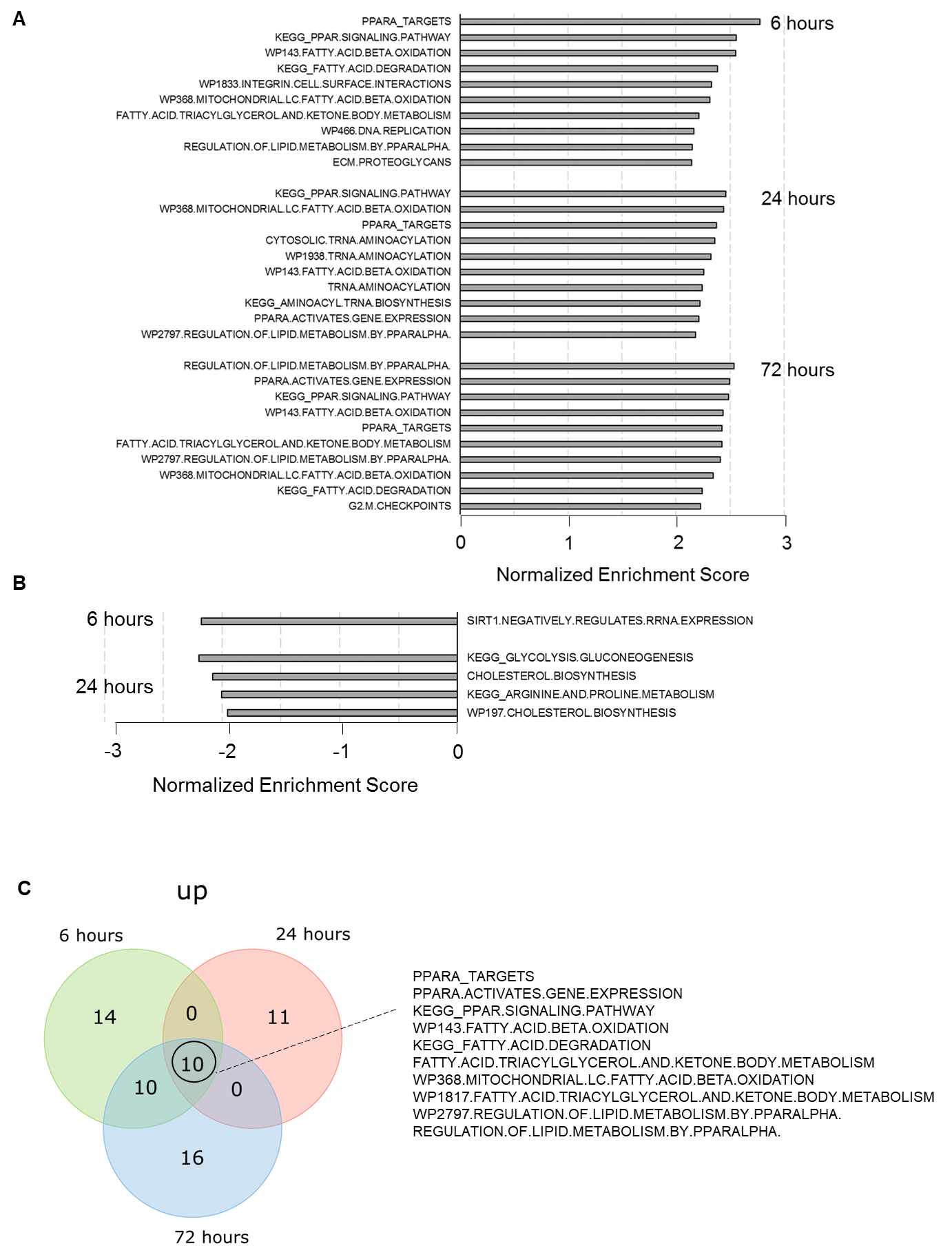
*

*Supplementary Fig. 7. Gene sets induced or repressed upon 6, 24 or 72 h exposure to PFOA in HepaRG cells. A and B) The top 10 most strongly induced (A) or repressed (B) gene sets in HepaRG cells in response to a 6, 24 or 72 h exposure were determined according to normalized enrichment score (NES) and statistical significance (FDR q-value <0.05) obtained with the gene set enrichment analysis (GSEA). Only 1, 4 and 0 gene sets were repressed upon exposure for 6, 24 or 72 h exposure, respectively. C) Venn diagram showing number of gene sets upregulated upon 6, 24, or 72 h exposure, presenting the gene sets commonly affected upon the three time points. No overlapping repressed gene sets were observed upon 6, 24, or 72 h exposure. Only gene sets were included with an FDR q-value, i.e. q<0.05.*


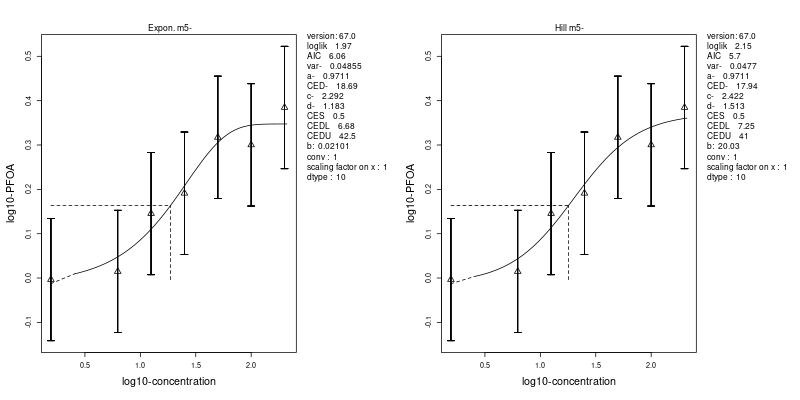


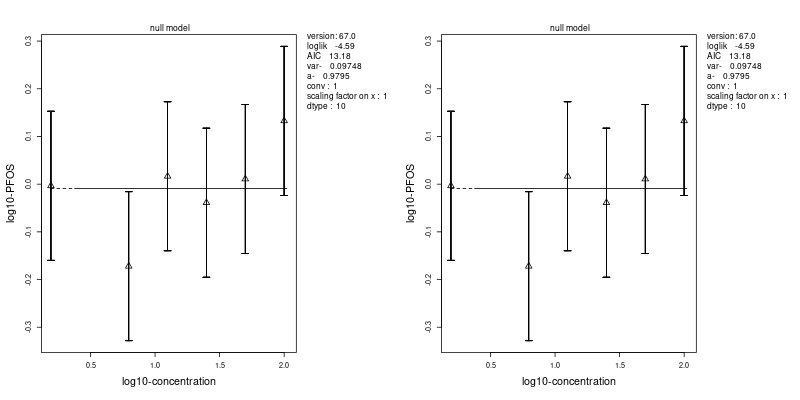


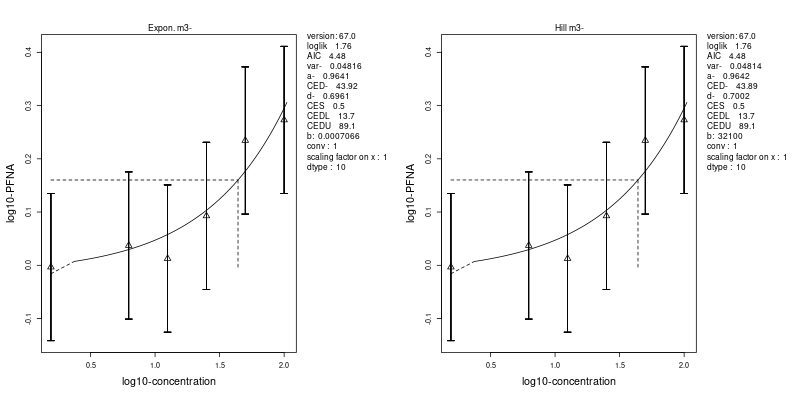


*Supplementary Fig. 8a. BMD analysis of changes in ANGPTL4 gene expression levels induced by exposure to PFOA (upper), PFOS (middle) and PFNA (lower), using two different curve fits (left: exponential, and right: Hill-function).*


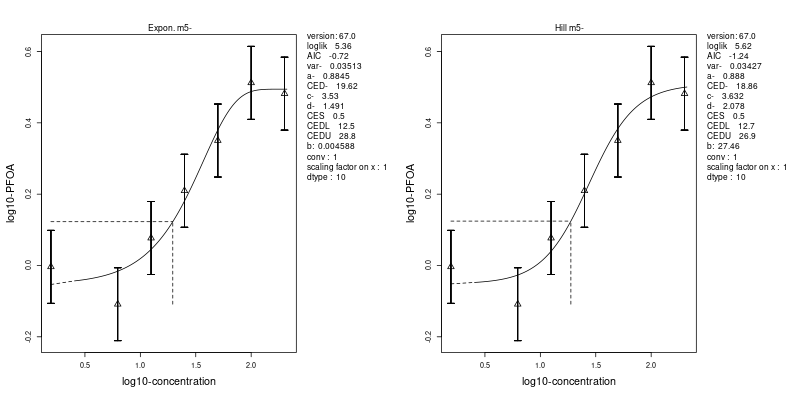


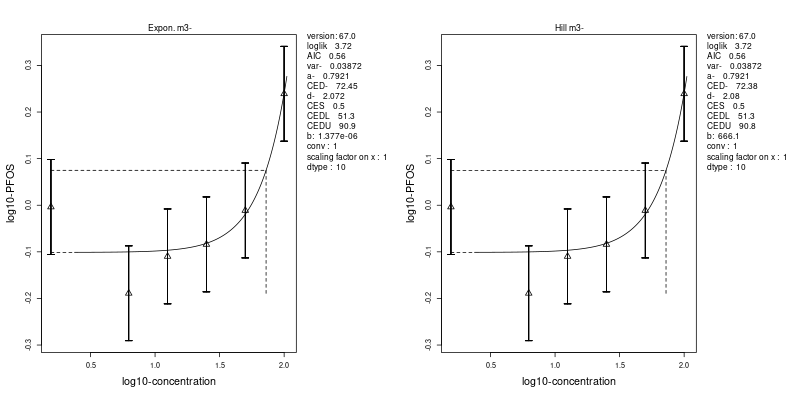


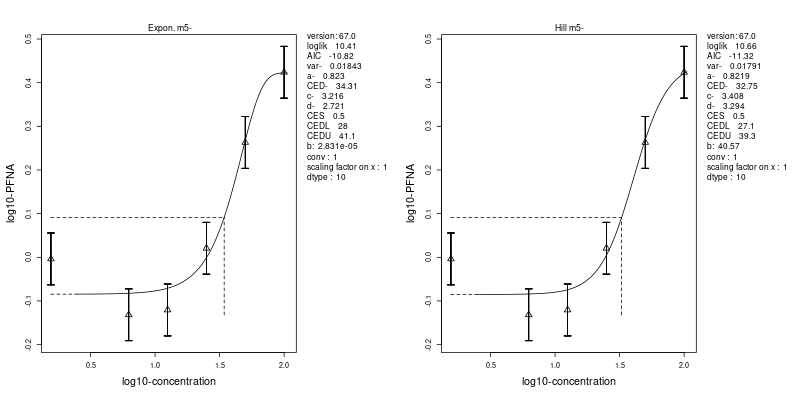


*Supplementary Fig. 8b. BMD analysis of changes in PDK4 gene expression levels induced by exposure to PFOA (upper), PFOS (middle) and PFNA (lower), using two different curve fits (left: exponential, and right: Hill-function).*


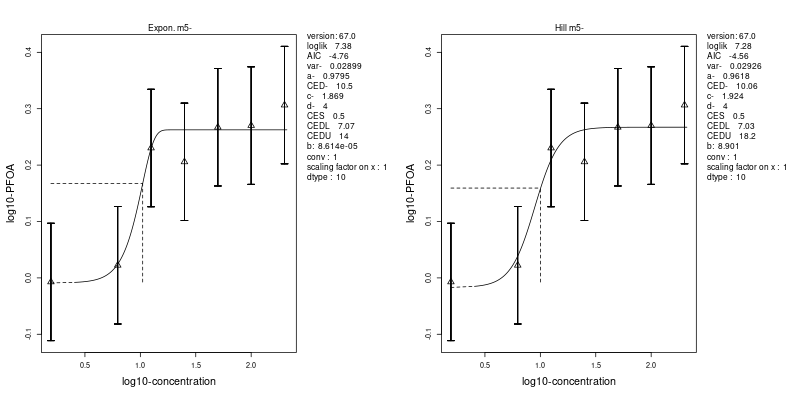


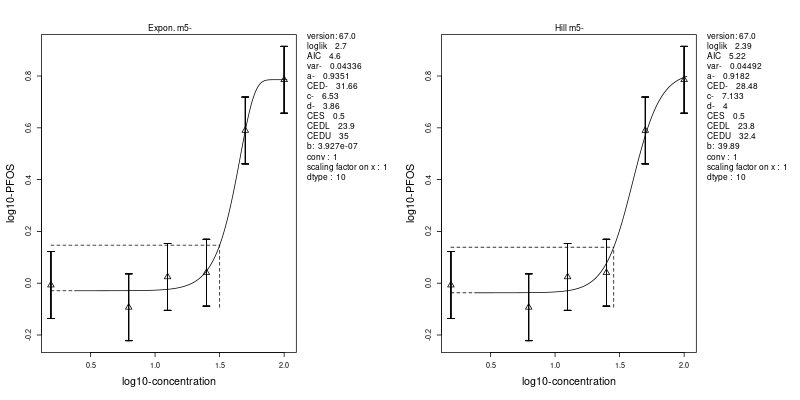


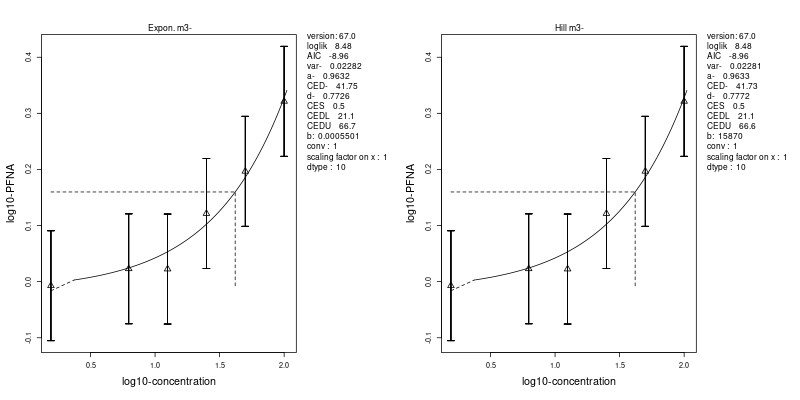


*Supplementary Fig. 8c. BMD analysis of changes in PLIN2 gene expression levels induced by exposure to PFOA (upper), PFOS (middle) and PFNA (lower), using two different curve fits (left: exponential, and right: Hill-function).*


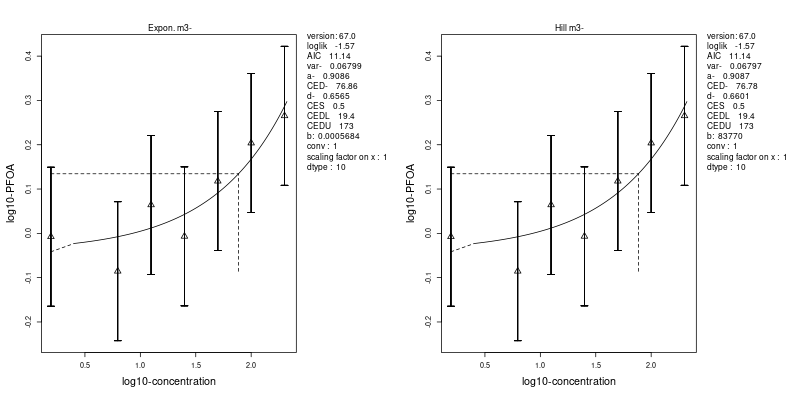


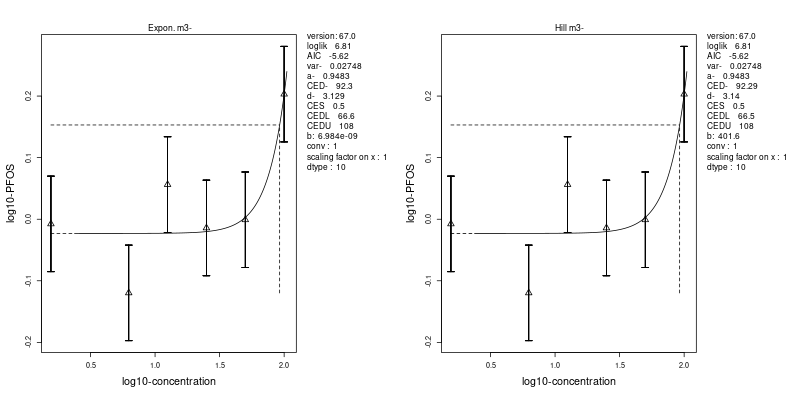


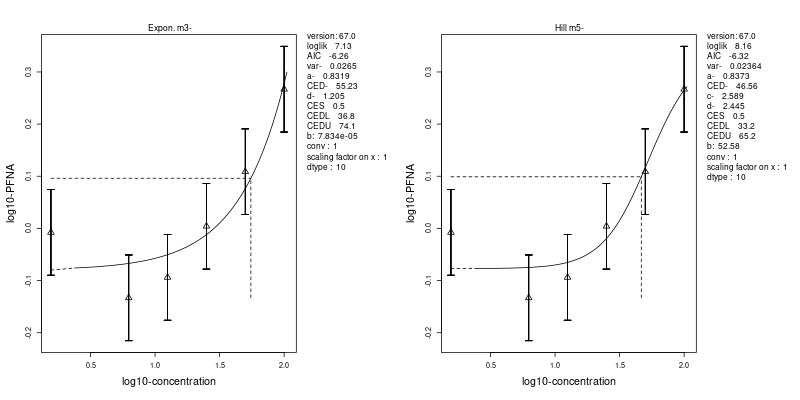


*Supplementary Fig. 8d. BMD analysis of changes in PLIN4 gene expression levels induced by exposure to PFOA (upper), PFOS (middle) and PFNA (lower), using two different curve fits (left: exponential, and right: Hill-function).*


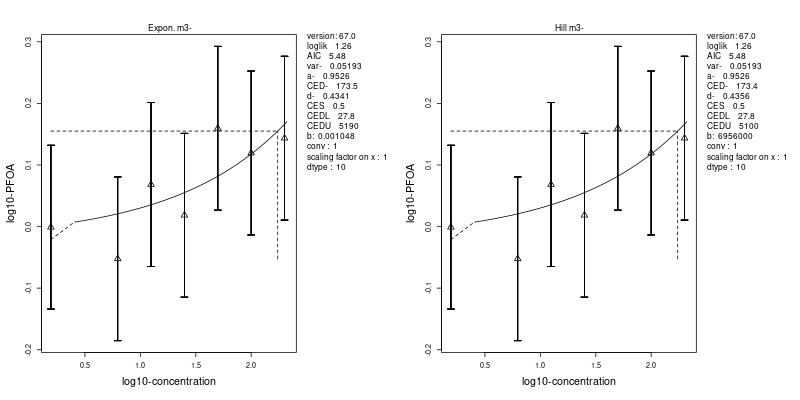


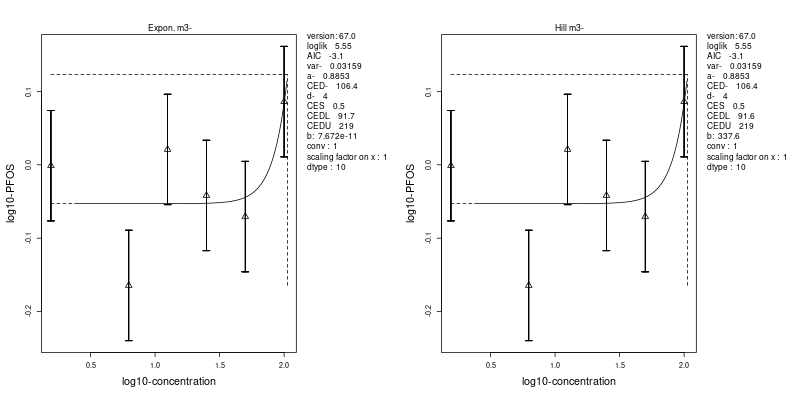


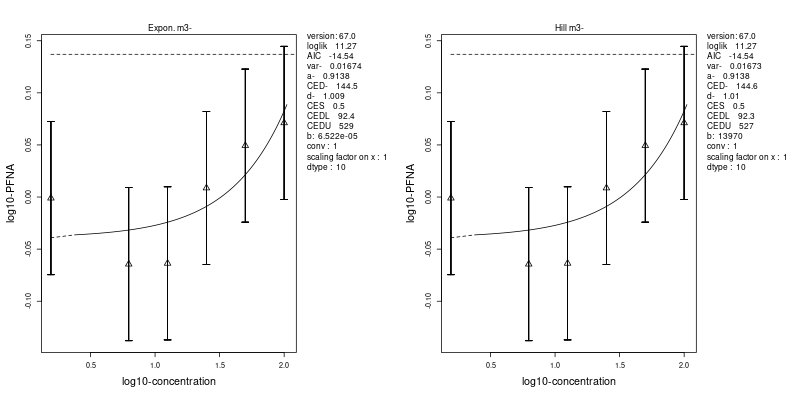


*Supplementary Fig. 8e. BMD analysis of changes in CPT1A gene expression levels induced by exposure to PFOA (upper), PFOS (middle) and PFNA (lower), using two different curve fits (left: exponential, and right: Hill-function).*


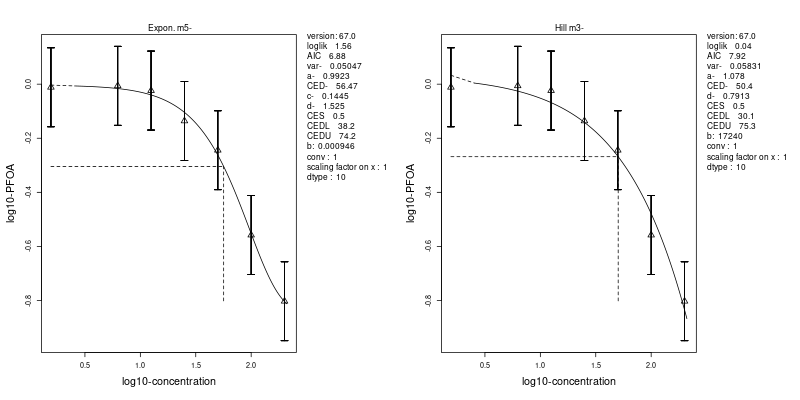


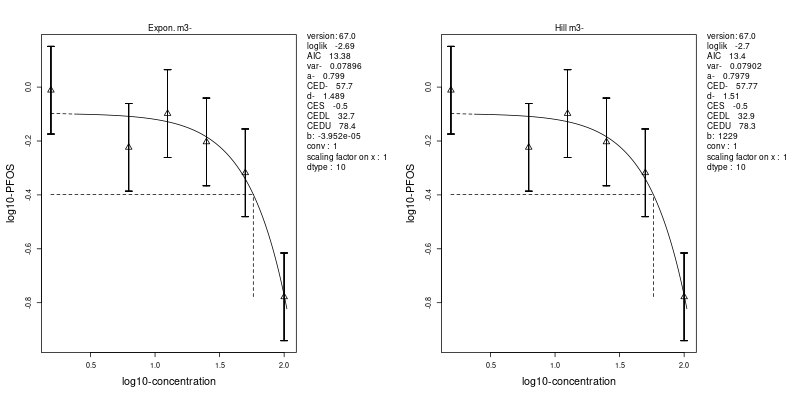


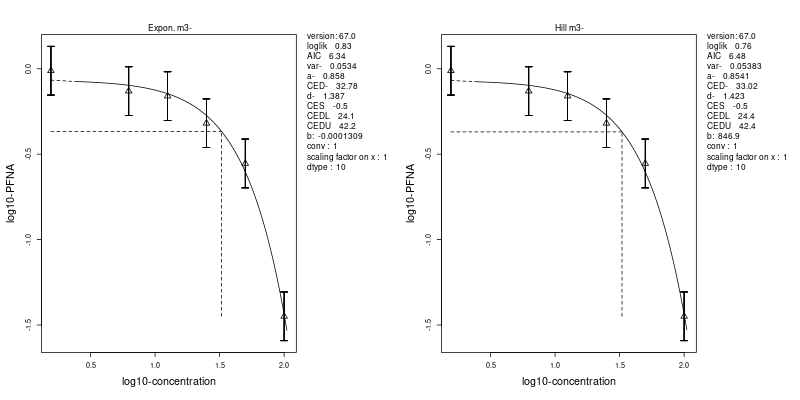


*Supplementary Fig. 8f. BMD analysis of changes in ADH4 gene expression levels induced by exposure to PFOA (upper), PFOS (middle) and PFNA (lower), using two different curve fits (left: exponential, and right: Hill-function).*


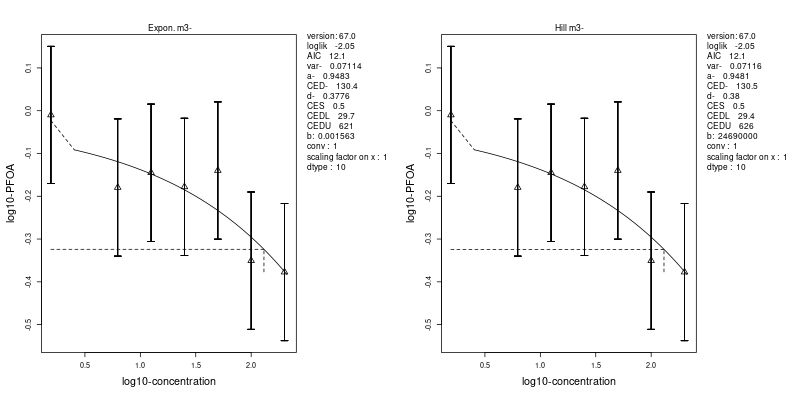

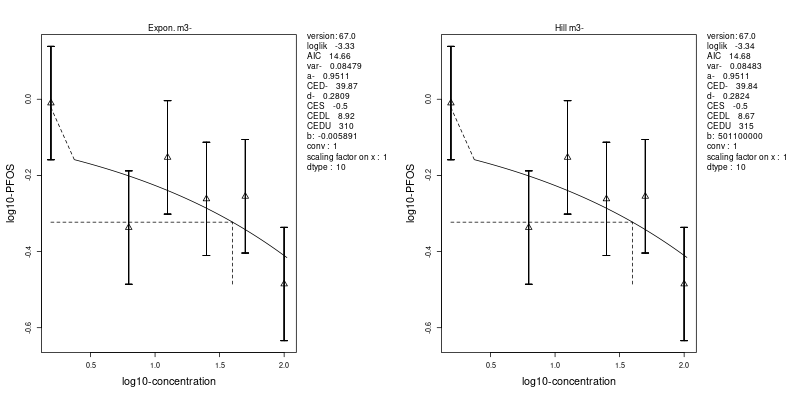

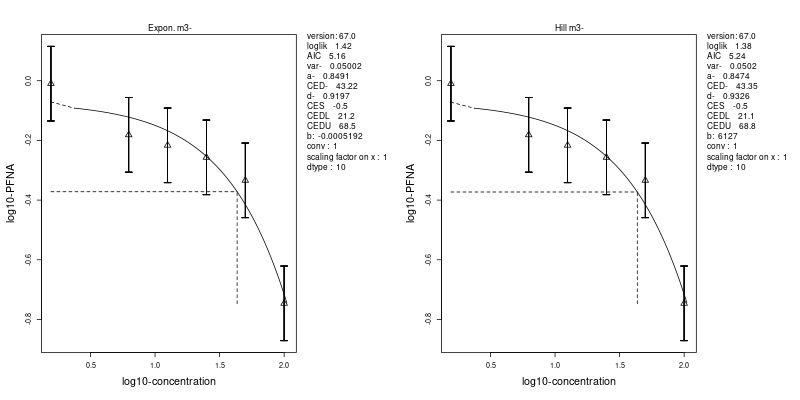


*Supplementary Fig. 8g. BMD analysis of changes in LSS gene expression levels induced by exposure to PFOA (upper), PFOS (middle) and PFNA (lower), using two different curve fits (left: exponential, and right: Hill-function).*


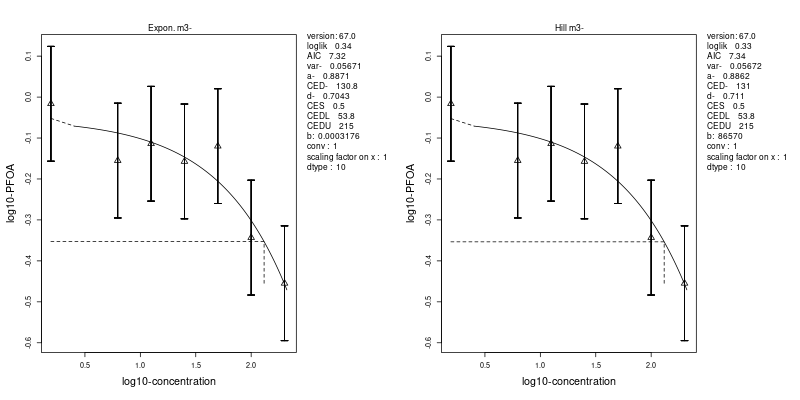

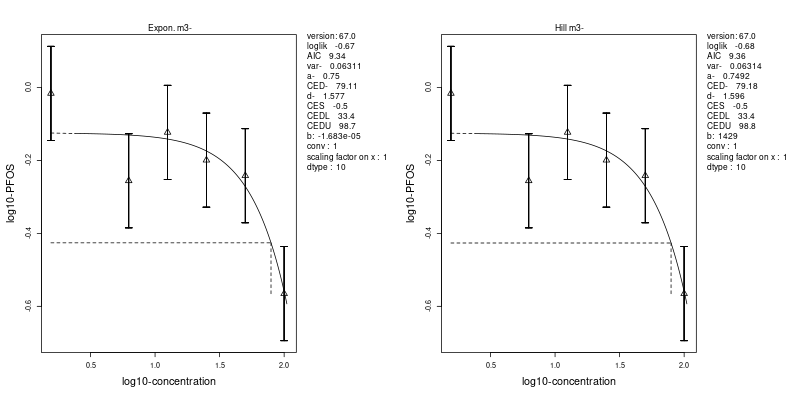

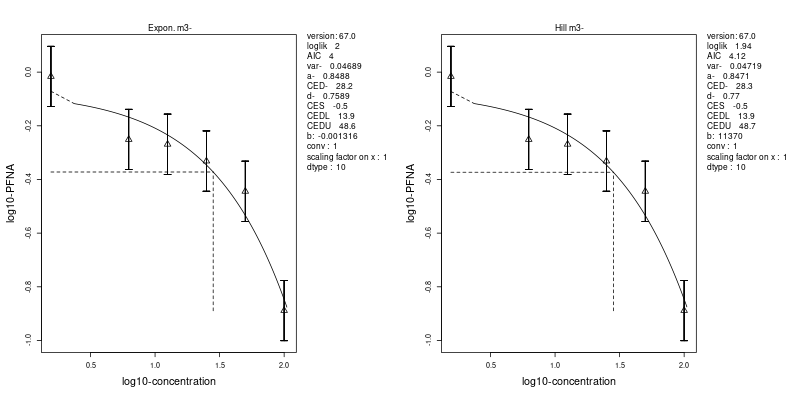


*Supplementary Fig. 8h. BMD analysis of changes in FDPS gene expression levels induced by exposure to PFOA (upper), PFOS (middle) and PFNA (lower), using two different curve fits (left: exponential, and right: Hill-function).*


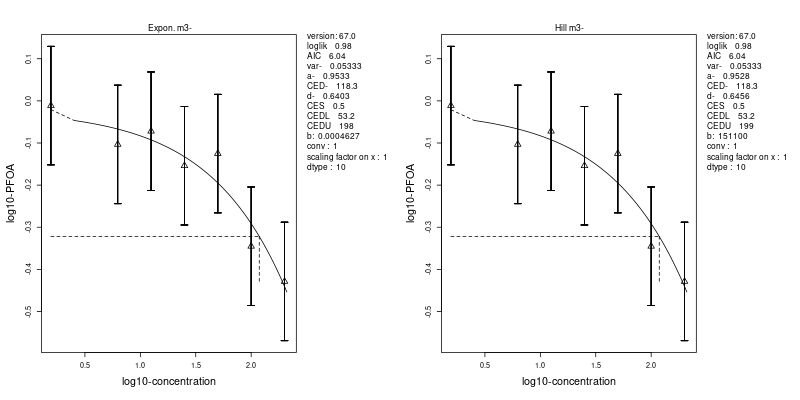


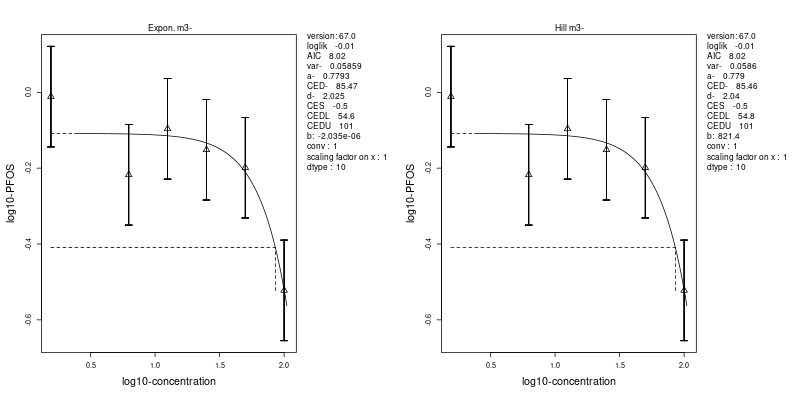


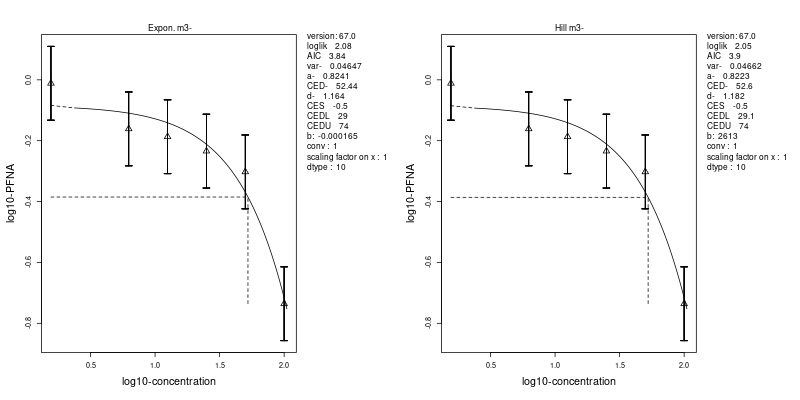


*Supplementary Fig. 8i. BMD analysis of changes in HMGCR gene expression levels induced by exposure to PFOA (upper), PFOS (middle) and PFNA (lower), using two different curve fits (left: exponential, and right: Hill-function).*


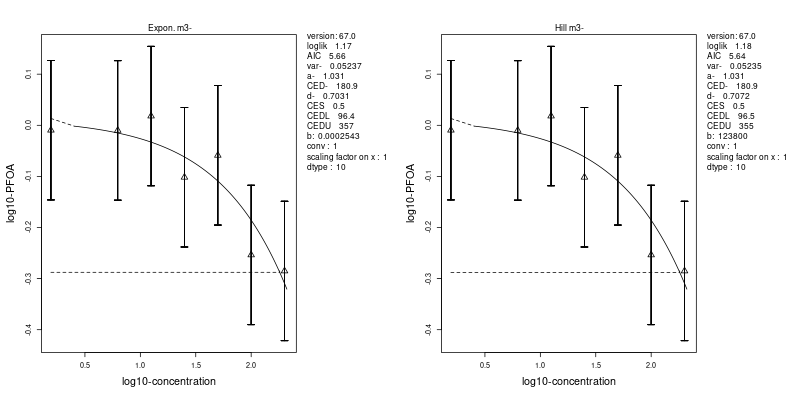


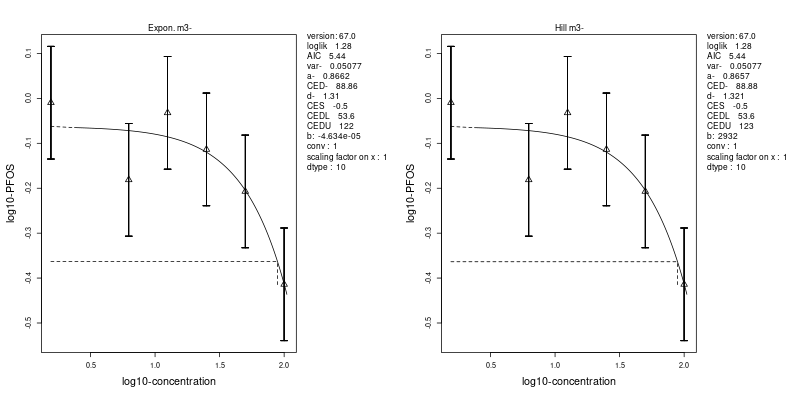


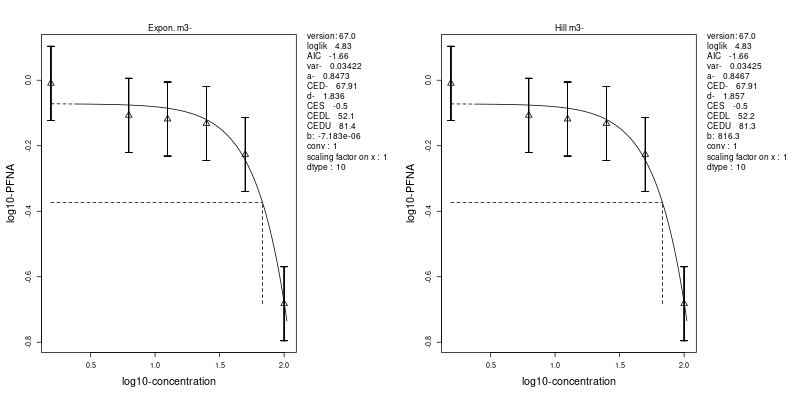


*Supplementary Fig. 8j. BMD analysis of changes in EBP gene expression levels induced by exposure to PFOA (upper), PFOS (middle) and PFNA (lower), using two different curve fits (left: exponential, and right: Hill-function).*


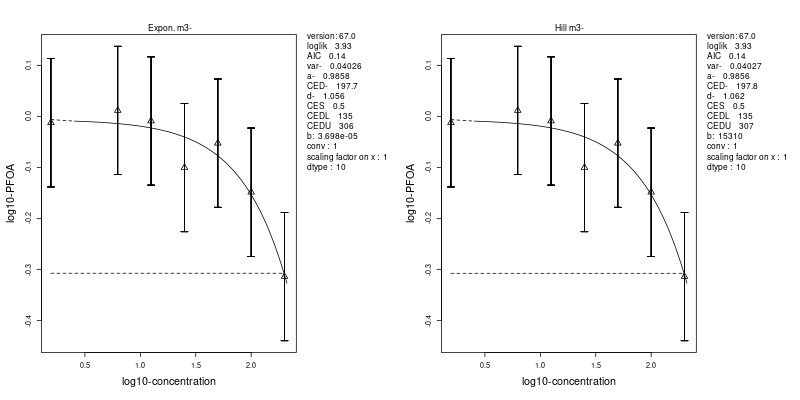


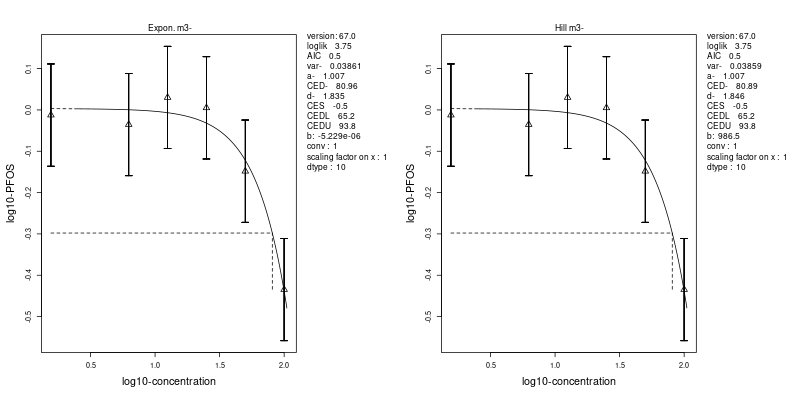


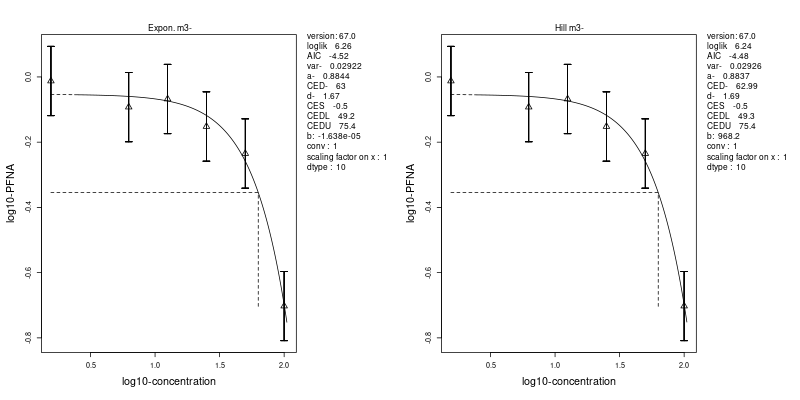


*Supplementary Fig. 8k. BMD analysis of changes in IDI1 gene expression levels induced by exposure to PFOA (upper), PFOS (middle) and PFNA (lower), using two different curve fits (left: exponential, and right: Hill-function).*


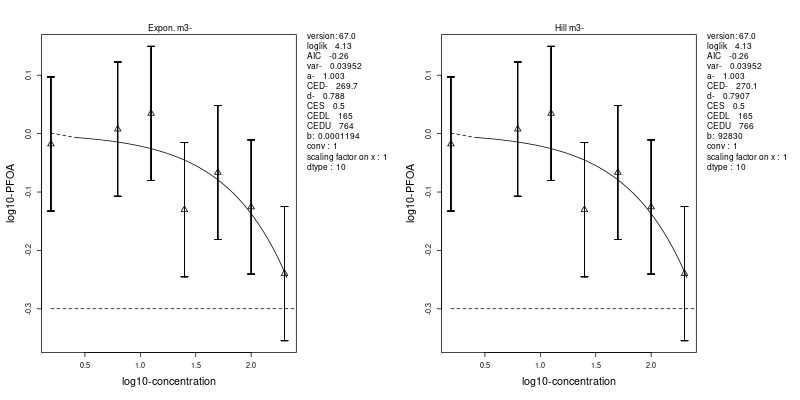


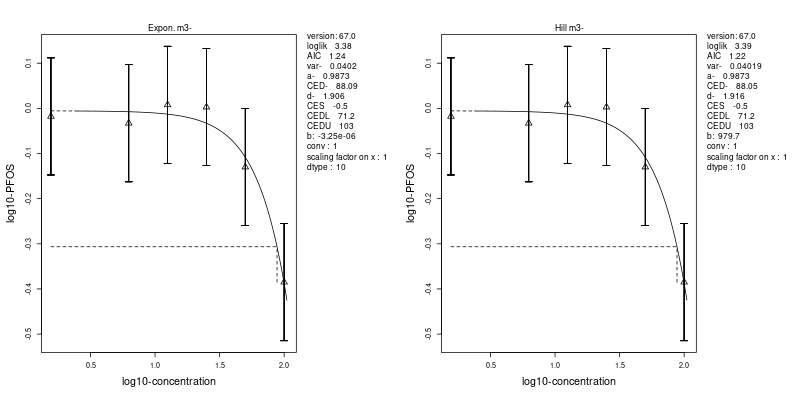


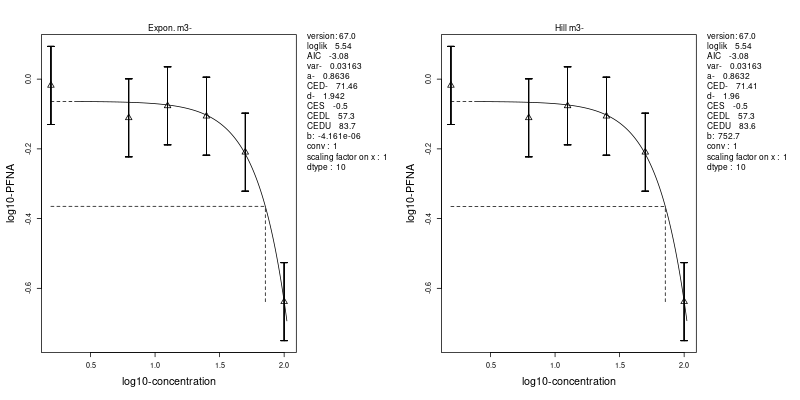


*Supplementary Fig. 8l. BMD analysis of changes in ACAT2 gene expression levels induced by exposure to PFOA (upper), PFOS (middle) and PFNA (lower), using two different curve fits (left: exponential, and right: Hill-function).*

*Supplementary Fig. 9. PFAS-induced changes in gene expression of genes related to SREBP signaling (Wikipathway WP1982).*
